# Can Focusing on One Deep Learning Architecture Improve Fault Diagnosis Performance?

**DOI:** 10.1021/acs.jcim.4c02060

**Published:** 2025-01-30

**Authors:** João G. Neto, Karla Figueiredo, João B. P. Soares, Amanda L. T. Brandão

**Affiliations:** † Department of Chemical and Materials Engineering, Pontifical Catholic University of Rio de Janeiro, 225, Marquês de São Vicente Street, Gávea, Rio de Janeiro, RJ 22451-900, Brazil; ‡ Department of Computer Science, Rio de Janeiro State University, 524, Rector João Lyra Filho Pavilion, sixth floor, Maracanã, Rio de Janeiro, RJ 20550-013, Brazil; ¶ Department of Chemical Engineering, University of Alberta, 9211, 116 Street, Edmonton, Alberta T6G 1H9, Canada

## Abstract

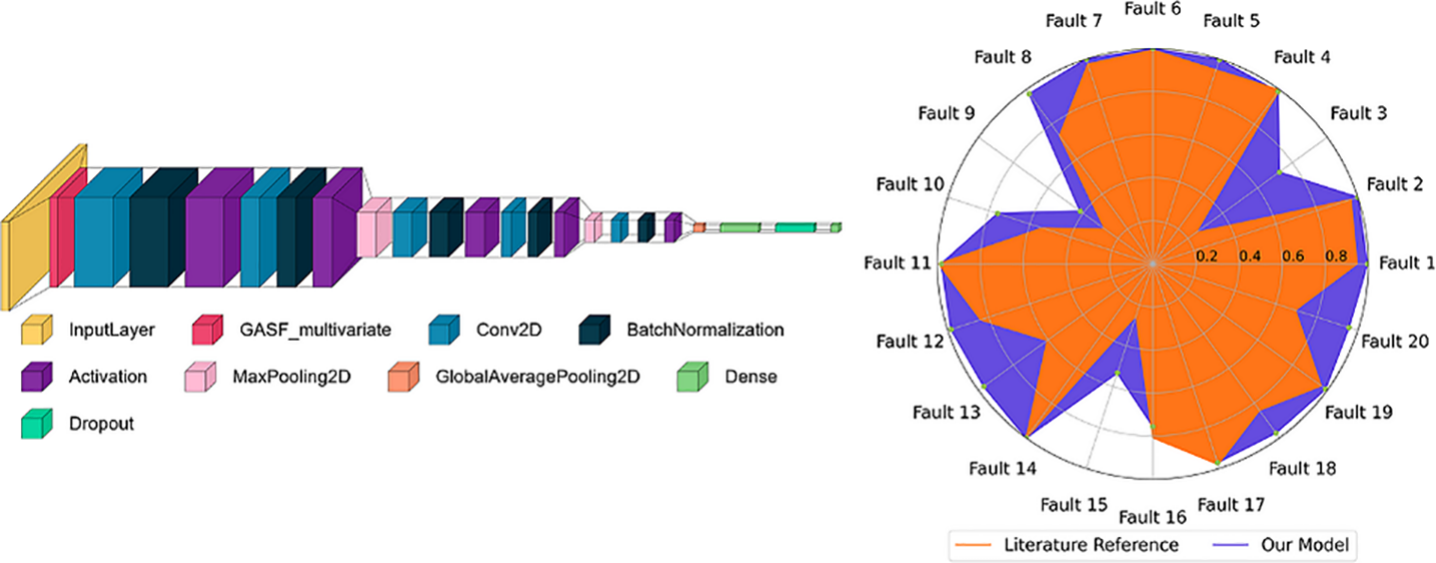

Machine learning approaches often involve evaluating a wide range of models due to various available architectures. This standard strategy can lead to a lack of depth in exploring established methods. In this study, we concentrated our efforts on a single deep learning architecture type to assess whether a focused approach could enhance performance in fault diagnosis. We selected the benchmark Tennessee Eastman Process data set as our case study and investigated modifications on a reference convolutional neural network-based model. Results indicate a considerable improvement in the overall classification, reaching a maximum average F1-score of 89.85%, 7.47% above the baseline model, which is also a considerable improvement compared to other performances reported in the literature. These results emphasize the potential of this focused approach, indicating it could be further explored and applied to other data sets in future work.

## Introduction

In machine learning studies, it is common practice to evaluate multiple models, given the vast array of available and emerging architectures. For instance, Neural Architecture Search can be used to automate the design of optimal neural networks, minimizing human involvement.^1−3^ However, when adopting Deep Learning (DL) alternatives, high computational costs and long processing times can limit architecture exploration. Since no single model consistently provides an optimal solution, we focused our efforts on investigating a single type of DL architecture to address the question: can this focused approach improve performance in fault diagnosis?

Observable deviations from designed operating conditions often precede accidents in chemical processes, providing early indicators of potential failures. These abnormal behaviors are related to a series of process failures usually triggered by a sensor, actuator, or another specific system fault.^4,5^ Pinpointing the original fault is not an evident task depending on the industrial plant’s complexity, nonlinearity, and process dynamics. Therefore, Fault Detection and Diagnosis (FDD) is the field of study dedicated to detecting anomalous system behaviors and diagnosing the root fault, which is responsible for the aforementioned condition.^6−8^

FDD is of extreme importance in chemical processes to ensure personnel safety, protect the environment, prevent costly shutdowns, and mitigate risks associated with abnormal situations, which can compromise operational stability and lead to cascading system failures.^4,9^ Modern chemical plants are highly complex, nonlinear, and dynamic, making FDD particularly challenging.^7,10,11^ Traditional model-based methods often become impractical due to their dependence on expert knowledge and difficulty in accurately describing such intricate processes.^12^ In contrast, data-driven approaches can use real-time monitoring and historical process data, aligning with Industry 4.0 advancements to provide scalable, robust, and adaptable solutions.^13,14^ To address these challenges, we focused on enhancing fault diagnosis performance through a data-driven technique applied to the Tennessee Eastman Process data set.^15^

The TEP plays a crucial role in FDD due to its realistic simulation of complex chemical processes. Its importance stems from its ability to provide a controlled yet intricate environment where various fault scenarios can be systematically introduced and analyzed. TEP allows researchers and practitioners to develop and test advanced FDD algorithms in a setting that mimics real-world challenges without the associated risks and costs of working with actual industrial systems. The TEP’s comprehensive range of fault types, including operational disturbances and sensor failures, provides a valuable testbed for evaluating the effectiveness of different diagnostic approaches and refining methodologies to enhance their robustness and accuracy. From a practical standpoint, this case study closely resembles actual industrial processes, which are often complex, costly, and sensitive to failures.^16−19^ For these reasons, we selected it as the case study for our investigation.

Numerous studies have investigated methods to improve performance of fault detection and diagnosis systems.^9,20,21^ Some approaches use Recurrent Neural Networks to process the time series, such as Long Short-Term Memory (LSTM) networks for FDD modeling.^22,23^ On the other hand, others focus on enhancing data representation through transformations, such as wavelet transforms combined with support vector machine classifiers.^24^

Recent advancements in intelligent fault diagnosis emphasize integrating deep learning and domain-specific adaptations to enhance model robustness and trustworthiness. Sun et al. introduced a domain adaptation-based method through one-dimensional convolutional autoencoders, achieving high diagnostic accuracy and flexibility across multiple operational conditions.^25^ Xie et al. proposed a unified out-of-distribution (OOD) detection framework combining class-wise outlier detection with supervised learning using a custom loss function composed of cross-entropy loss and contrastive learning loss, improving the reliability of prognostics and health management systems in renewable energy applications.^26^ Zhang et al. developed a deep ensemble approach guided by max-consistency and min-similarity to address diagnostic uncertainty and enhance fault detection in industrial systems.^27^ These studies focus on OOD detection, transfer learning, and ensemble strategies in achieving promising perspectives for adaptive fault diagnosis, providing a foundation for incorporating similar methodologies into broader industrial contexts.

In our work, we focused on investigating the Gramian Angular Fields (GASF) approach^28^ for time series transformation, followed by Convolutional Neural Networks (CNNs) for feature learning and fault classification based on the study performed by Sun and Ren.^29^ GASF transforms time series into a structured, 2D matrix representation, enabling the application of CNNs to detect patterns across both temporal and variable dimensions. By allowing simultaneous cross-variable analysis through multichannel inputs, this approach provides a robust framework for identifying intricate fault interactions, even when their timing and expression within the system are highly complex and variable.

We focused on CNNs due to their ability to efficiently process transformed time series data and their capacity to learn features from data. This practical balance between performance, computational cost, and feature learning aligns well with our research’s data set characteristics and goals. While alternative deep learning approaches remain promising and viable options, our decision reflects the alignment of CNNs with the scope of this work.

Following their proposed methodology, we built a baseline model and investigated a series of data preprocessing and architecture modifications to search and evaluate their effects on model performance. Our investigation resulted in a model with an improved F1-score of 89.85% at a shorter or similar process observation window compared to existing literature.

This paper is organized as follows: The Data Set Description section provides overviews of the Tennessee Eastman Process data set and discusses challenges associated with time series analysis. In the Fundamentals of the Model Architecture section, we explain Convolutional Neural Network architectures, show relevant works within the Fault Detection and Diagnosis context, and detail the Gramian Angular Summation Fields data transformation. The Methodology section outlines the data pretreatment steps, describes the baseline model architecture, details the training parameters used, and specifies the modifications investigated in our study. The Results and Discussion section comprehensively evaluates our approach, addressing its limitations and suggesting potential improvements. Finally, the Conclusion section summarizes our findings and outlines directions for future research.

## Data Set Description

This section introduces the Tennessee Eastman Process data set, a widely recognized fault detection and diagnosis research benchmark. The data set provides comprehensive multivariate time series data for evaluating and developing our fault diagnosis models.

### Tennessee Eastman Process

**Figure 1:**
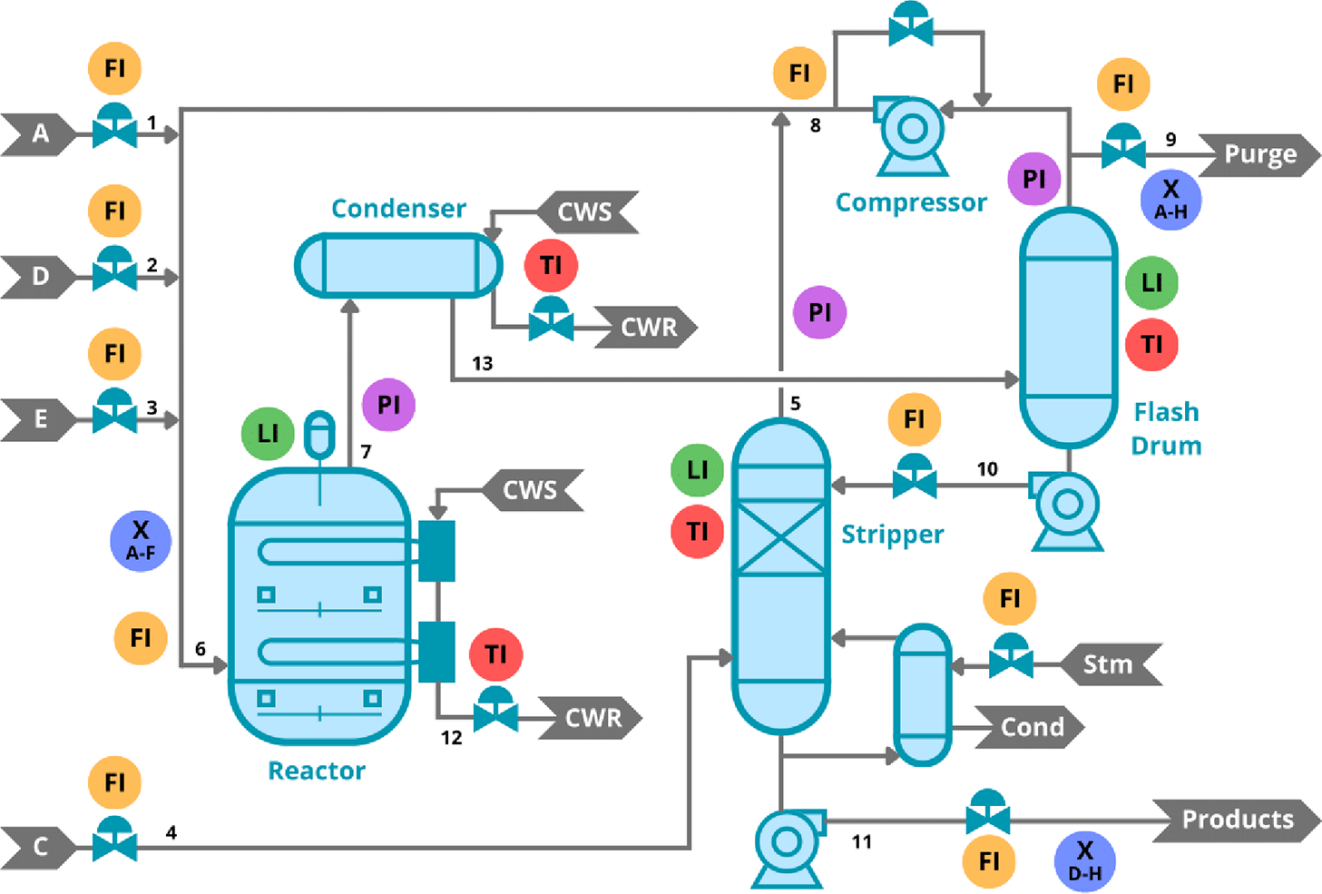
Tennessee eastman process diagram. Adapted from Piebalgs.^30^

The Tennessee Eastman Process (TEP) is a widely recognized simulation of a chemical production process used extensively in process control and fault detection research. Developed by Downs and Vogel in 1993,^15^ the TEP models a complex chemical plant with two products involving several interconnected reactions, as shown in Figure 1. The process operates under varying conditions and includes key features, such as reaction kinetics, heat exchanges, and multiple process disturbances. Its complexity, with numerous operational variables and potential fault scenarios, provides a robust platform for testing and evaluating various control strategies and FDD methods.

### Data and Software Availability

The version of the TEP raw data we selected for our study is available on the Kaggle platform.^31^ It includes four reactants (A, C, D, and E), an inert compound (B), a byproduct (F), and two targeted products (G and F). It contains 52 process variables, of which 12 are manipulated variables, 22 are process measurements, and 18 are component analyses. In addition to normal behavior, this version contains 20 faults as described in Table 1. Faults 5, 8, 9, 10, 15, and 16 have been reported as challenging to diagnose.^32,33^ It is important to comment that some databases include a possible 21st fault in the TEP, but the data set we selected consisted of the original 20 faults.

**Table 1:** List and Description of Faults of the TEP

no.	description	type
1	A/C feed ratio, B composition constant (stream 4)	Step
2	B composition, A/C feed ratio constant (stream 4)	Step
3	D feed temperature (stream 2)	Step
4	Reactor cooling water inlet temperature	Step
5	Condenser cooling water inlet temperature	Step
6	A feed loss (stream 1)	Step
7	C header pressure loss-reduced availability (stream 4)	Step
8	A, B, and C feed composition (stream 4)	Random
9	D feed temperature (stream 2)	Random
10	C feed temperature (stream 4)	Random
11	Reactor cooling water inlet temperature	Random
12	Condenser cooling water inlet temperature	Random
13	Reaction kinetics	Slow drift
14	Reactor cooling water value	Sticking
15	Condenser cooling water value	Sticking
16	Unknown	Unknown
17	Unknown	Unknown
18	Unknown	Unknown
19	Unknown	Unknown
20	Unknown	Unknown

Since the TEP data set exhibits intricate multivariate time series characteristics, traditional statistical techniques may not fully capture the temporal dependencies and complex relationships within such data. As we transition to the next section, we consider these potential limitations and explore the need for alternative approaches to analyze and interpret this type of data effectively.

### Limitations of Multivariate Time Series Analysis

Statistical techniques such as Regression Analysis and Pearson Correlation^34^ are commonly employed for feature screening by characterizing the linear relationships between pairs of variables. These traditional methods are widely used because of their simplicity and effectiveness in many scenarios. However, when applied to serialized or time-dependent data, they can lead to significant information loss or misleading conclusions, which may occur because they do not account for the temporal structure of the data, which is a critical aspect of time series analysis.^35^

In the time series context, alternative methods such as autocorrelation and partial autocorrelation analysis provide valuable insights. These techniques help to understand the influence of time lags on feature behavior by examining how past values of a series affect its current state.^36^ However, autocorrelation and partial autocorrelation mainly capture linear relationships and rely on the assumption that statistical properties remain constant over time. This can be overly restrictive in real-world data, where nonlinearities, trends, or structural breaks often occur. Moreover, these methods are limited to univariate analysis and do not account for interactions between multiple features.

To extend the analysis, exploring time-lagged cross-correlation can help assess relationships between features across different time delays.^37^ Although this method offers insights into the relationships between variables, it can become computationally prohibitive as the number of features and the length of the observation window increase exponentially. This scalability issue poses a significant challenge when dealing with large data sets typical of real-world applications. However, how could we address this challenge?

We opted to retain all variables and investigate a type of model capable of feature learning: the process in which a machine learning model automatically extracts meaningful and relevant features from input data to improve predictions based on high-dimensionality and complex data.

## Fundamentals of the Model Architecture

This section focuses on the model architecture we investigated to address our fault diagnosis challenge. We start with Convolutional Neural Networks, highlighting their role in feature extraction and classification tasks. Then, we introduce Gramian Angular Summation Fields (GASF), which serves as a data transformation technique that we integrated into our models. By exploring this architecture in detail, we aim to provide a comprehensive understanding of its contributions to enhancing model performance and handling complex time series data.

### Convolutional Neural Networks

A Convolutional Neural Network (CNN) is a type of deep learning model particularly well-suited for processing data with a grid-like structure, such as images.^38^ It processes the spatial hierarchies in data using convolutional layers, which apply filters to input data to extract features, such as edges, textures, or complex patterns. Mathematically, a convolution operation involves sliding a filter over the input data, computing the dot product between the filter and the portion of the input it covers. The result is a feature map that highlights the presence of specific features in the input. This process can be repeated through multiple convolutional layers, progressively extracting higher-level features from the data.^39^

**Figure 2:**
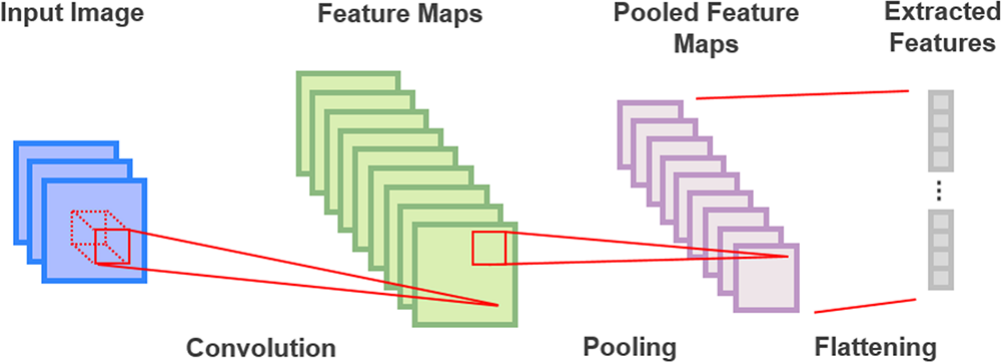
Illustration of feature learning in CNNs.

Additionally, pooling layers are often used after convolutional layers to reduce the feature maps’ spatial dimensions, which helps decrease the computational load and control overfitting.^40^ There are different types of pooling operations such as max pooling, which selects the maximum value within a defined region of the feature map, or average pooling, which computes the average value. Through this combination of convolution and pooling, CNNs can effectively capture and summarize the essential patterns in the input data. This process is illustrated in Figure 2.

At the end of the feature learning process, the extracted features typically go through a fully connected layer or classifier, which maps these features to the final output, such as class labels in classification tasks.^38^

Traditional CNN architectures, while powerful, face limitations primarily related to computational cost, depending on their structure. CNNs are computationally intensive, especially as the network depth and the number of filters increase, which can lead to significant resource demands, especially during training. Additionally, using CNNs with other data types, such as sequences or graphs, requires data transformation or specialized architecture variations to handle such inputs effectively.^32,33,41^

The decision to focus on CNNs in this study, rather than exploring other deep learning models like Long Short-Term Memory (LSTM) networks, Autoencoders (AE), or Transformers, was influenced by the characteristics of the data set and the specific advantages of using Gramian Angular Fields alongside CNNs.

CNNs are particularly well-suited to analyzing 2D representations of time series data, such as GASF, due to their ability to detect spatial patterns. This capacity for feature learning allows CNNs to automatically extract relevant patterns and relationships from data without relying heavily on handcrafted feature engineering. CNNs excel at identifying both local and global dependencies within transformed time series data, enabling robust detection of complex fault interactions.^28,39^

LSTM networks are a compelling choice for tasks involving raw time series data, as they excel in capturing sequential dependencies.^42^ While LSTMs have been explored in this context, their feature learning capabilities are typically centered on temporal patterns of the individual time series, having their cross-relationships captured in the deeper layers of the network. On the other hand, the matrix structure provided by GASF allows CNN to try capturing these relationships from the beginning of the network.

AE are effective for dimensionality reduction and unsupervised anomaly detection, but their use in supervised classification tasks like fault diagnosis often requires additional layers or hybrid architectures.^43^ In contrast, the CNN structure allows for direct training from input to label, with feature extraction as part of the learning process.

With their self-attention mechanisms, transformers are increasingly popular for time series analysis and offer notable flexibility in modeling complex relationships.^44^ However, their inherent complexity and large model sizes can pose challenges, especially with constrained computational resources. While these architectures are highly expressive, their design may introduce added complexity for tasks that models like CNNs can potentially address.

While each alternative method offers unique strengths and could be explored in future works, CNNs’ ability to automatically learn features from spatially transformed data makes them particularly compelling in the context of fault diagnosis. Combined with their computational efficiency and compatibility with GASF, CNNs provided a robust framework for evaluating fault diagnosis performance while minimizing the need for extensive manual feature engineering.

Several recent publications have utilized CNNs for Fault Detection and Diagnosis in the Tennessee Eastman Process context. The studies briefly presented next were selected explicitly for comparison after our study to assess how our model performs relative to the current state-of-the-art.

E Souza et al. explored various CNN topologies.^45^ Their approach involved stacking different time series variables into a single matrix. While this technique facilitates the application of conventional CNN methods, it limits the ability of the filters to consider all process variables simultaneously.

Tao et al. proposed a Triage-based Convolutional Neural Network with a 1D-CNN as backbone.^46^ In their investigation, They considered 15 of the 20 TEP faults.

Ren et al. explored an ensemble-like approach, utilizing multiple 2D transformation techniques that were stacked and fed into a traditional CNN model.^47^ Simultaneously, the original time series data were processed through a 1D-CNN architecture. The final classification was a combination of the output of both models. Unfortunately, this study does not include some of the most challenging faults in its investigation.

Sun and Ren developed a fault diagnosis approach that converts multidimensional temporal data into multichannel 2D images using Gramian Angular Fields, followed by multiscale convolution modules for spatiotemporal feature extraction (GASF-MSNN).^29^ Their architecture was benchmarked against various models, including Gramian Angular Summation Fields–CNN (GASF-CNN), traditional CNN with series stacking, 1D-CNN, and LSTM networks. Although their results indicate that the GASF-MSNN model outperforms other approaches, the findings are limited by the scope of their investigation, leaving significant room for further exploration given the wide range of possible hyperparameter variations across the architectures.

Notably, their GASF-MSNN performed comparably to their GASF-CNN, prompting us to focus our investigation on the GASF-CNN due to its innovative data transformation technique and well-established classification architecture.

### Gramian Angular Summation Fields

The Gramian Angular Summation Fields (GASF) transformation is a technique used to convert time series data to account for variations for all possible time lag combinations within the series. This transformation is a part of the broader family of Gramian Angular Fields (GAF) Proposed by Wang and Oates.^28^ The key idea behind GASF is to use the polar coordinate system to encode the temporal dynamics of time series data into a matrix format.

The GASF transformation is mathematically computed by first scaling the time series data using a min-max normalization (*x̃*_*i*_). This step ensures that the data points can be mapped to angles in the polar coordinate system.

Next, the normalized values *x̃*_*i*_ are transformed into angular values theta using the arccosine function as shown in eq 1.
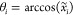
1

We obtain the GASF matrix by calculating the pairwise summation of the cosines of these angles, represented in eq 2.
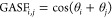
2

This transformation results in a symmetric matrix where each element represents the cosine of the sum of the angles corresponding to the normalized time series values. After a set of mathematical simplifications, obtaining the same GASF matrix directly from the normalized arrays is possible by performing the calculations shown in eq 3.
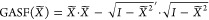
3where *X̃* is the array containing the normalized values of the time series sample and *I* is the identity matrix.

**Figure 3:**
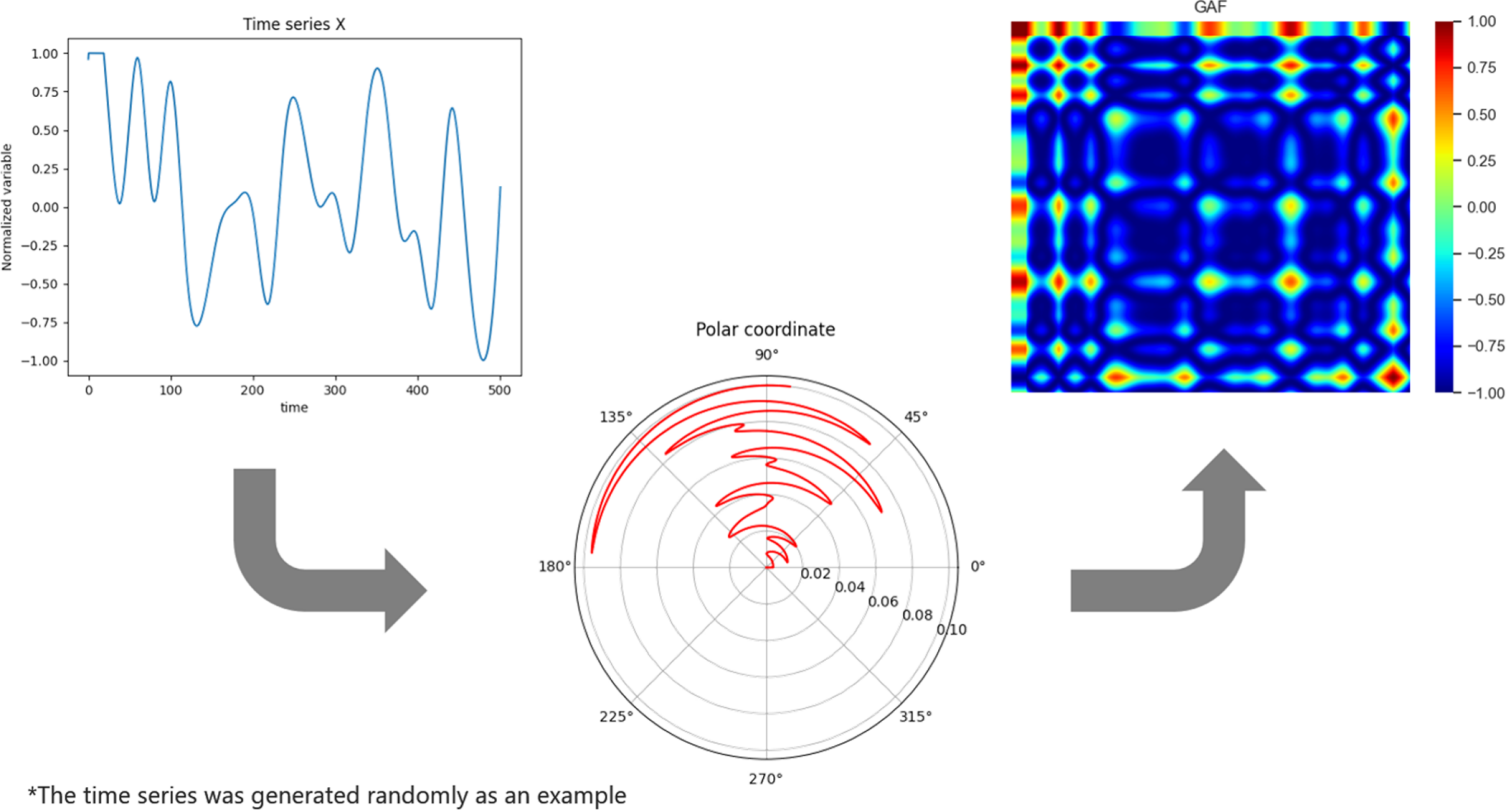
Illustration of a GAF type encoding for a general time series.

These transformations can be applied to multivariate time series, creating a matrix for each variable. This collection of matrices can be stacked and processed by CNNs and other image-based models for classification tasks. We illustrated this transformation process in Figure 3.

In the context of FDD, problems present unique challenges due to the unpredictable and complex ways faults manifest in multivariate time series data. Faults often arise from subtle, nonlinear interactions across multiple variables, and their expressions may vary temporally, making it difficult to pinpoint the exact variable or time window where anomalies occur.^6−8^

GASF transformation not only preserves the temporal sequence of data but also structures the information in a way that enhances the capabilities of Convolutional Neural Networks. CNNs are particularly effective at detecting spatial patterns in data. When applied to GASF-transformed matrices, they can search for fault patterns without being constrained to specific variables. Thus, they are especially advantageous in FDD, where anomalies may emerge in localized or dispersed regions of the time series.

Another critical advantage of GASF is its ability to represent multivariate data as multichannel input for CNNs. This setup enables the model to analyze interactions across all variables using filters. Kernels extract patterns that span both time (rows and columns of the matrices) and variable interactions (across channels) simultaneously.

Finally, combining GASF and CNNs enables a fault detection framework that accommodates systems with numerous variables. Knowing where faults might emerge is part of the model’s specialized feature extraction capabilities. It takes advantage of CNN’s inherent pattern recognition capabilities to identify anomalies wherever they occur, thus addressing the stochastic and distributed nature of fault expressions in complex systems.

## Methodology

This section outlines the methodology employed in our Fault Diagnosis model investigation. The process encompasses several critical stages, including data pretreatment, baseline model architecture, training hyperparameters, and investigated modifications.

We executed all data processing, model development, and evaluation tasks using Python. Specifically, we used the Pandas,^48^ TensorFlow,^49^ and Keras^50^ libraries for data manipulation and preprocessing, building and training the convolutional neural network models, and implementing custom layers and managing the model architecture.

In terms of hardware, processing was carried out using the i5–13600K CPU and 32 GB RAM, without the need for a dedicated GPU, which we consider an advantage as it shows the accessibility of our approach. Additionally, we used the tensorflow.data.dataframe module to improve data management during processing.

### Data Pretreatment

The original data set^31^ contains a total of 15,330,000 data samples, sampled every 3 min. This data set consists of 5,250,000 samples for training and 10,080,000 samples for testing. Given the long processing times and high computational costs associated with CNN models, we decided to perform random stratified subsampling on the training data. We then split the data into three distinct data sets that include 20 possible faults since our investigation focused on developing a diagnosis model.

Following the methodology of Sun and Ren,^29^ we used a window size of 20 data samples. This resulted in the following data sets: the training data set consisted of 36,600 windows (1800 per class), the validation data set included 9000 windows (450 per class), and the testing data set contained 5000 windows (250 per class). In contrast, Sun and Ren reported using training and testing data sets of 461 and 781 samples per fault, respectively and did not mention a validation set.

After splitting the data set, we normalized each variable based on the values from the training data. We capped all values within the training range for the validation and test data sets to prevent invalid numbers in subsequent calculations. We incorporated the Gramian Angular Summation Field (GASF) transformation into the model using a custom layer. This approach is advantageous because it reduces the extensive storage space required to precompute and save the transformed data for large data sets. By performing the GASF transformation within the model, we efficiently manage memory usage and storage requirements, as the transformation is computed on the fly during training and inference.

### Baseline Model Architecture

We adopted the GASF-CNN architecture proposed by Sun and Ren^29^ as our baseline model due to its strong performance, which showed less than a 1% difference in F1-score compared to their more complex GASF-MSNN model. This model employs a custom GASF layer to transform the time series data into a set of 52 matrices, which then serve as input for a series of convolutional and pooling layers dedicated to feature extraction. These specialized features are the input of dense layers responsible for fault classification.

**Figure 4:**
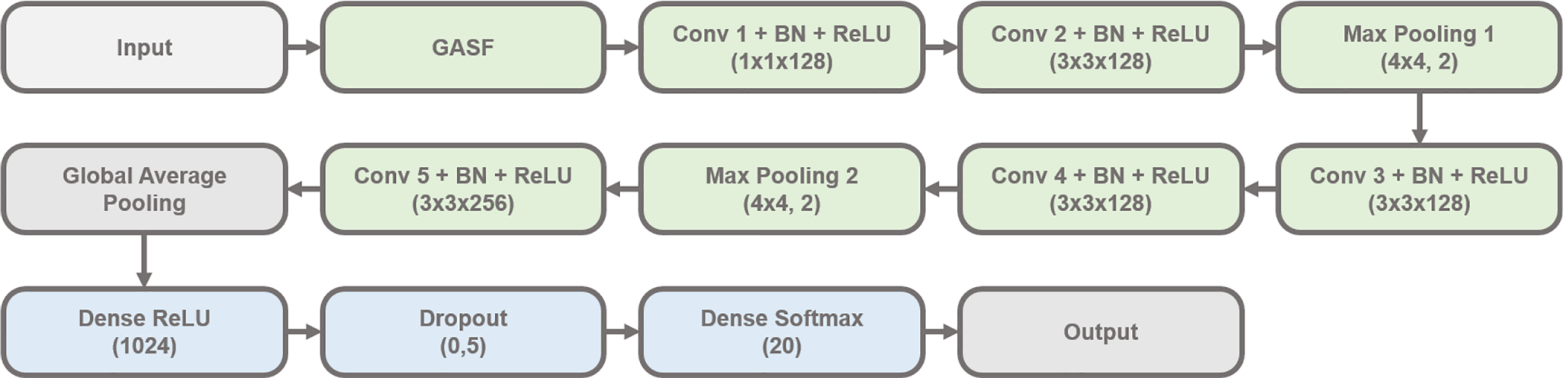
Baseline model architecture. Adapted from Sun and Ren.^29^

In order to enhance model generalization and reduce overfitting, batch normalization is applied between the convolutional layers, and a dropout layer is introduced between the dense layer and the softmax output layer. The reference authors did not specify the padding option used in the convolutional layers. However, in our implementation, we consistently set the padding to ‘same’ across all convolutional layers. This choice ensures that the output dimensions of the feature maps remain identical to the input dimensions, preserving the spatial resolution of the data across all layers. Figure 4 provides a detailed diagram of the architecture, including specifics such as the number of kernels and kernel sizes.

### Training Hyperparameters

In alignment with the methodology of Sun and Ren,^29^ we configured the batch size to 64 to optimize computational efficiency and model performance. We employed the Adam optimizer^51^ and used categorical cross-entropy^52^ as the loss function to address the multiclass nature of the fault diagnosis problem.

In order to mitigate overfitting, we implemented an EarlyStopping callback^50^ with a ‘patience’ argument of 15 epochs, monitoring performance on the validation set. The model’s weights were restored to their best state upon triggering early stopping. Furthermore, we set the maximum number of training epochs to 500 to ensure comprehensive model training.

**Figure 5:**
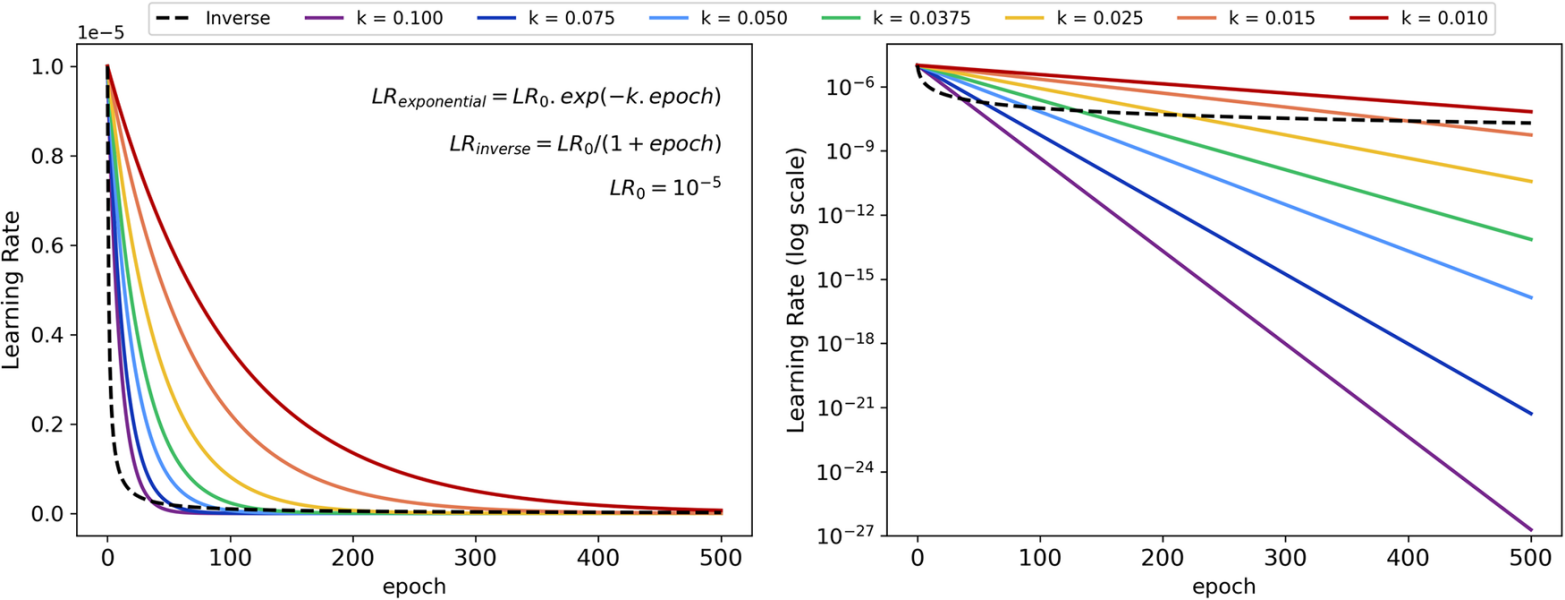
Investigated learning rate decay functions.

In their work, Sun and Ren^29^ do not mention the adopted learning rate, nor if they used any scheduler strategies. Therefore, in our initial investigation consisted we evaluated different learning rate decay functions as shown in Figure 5 applied to the baseline model.

### Investigated Modifications

In this section, we present the modifications implemented to enhance the performance of the baseline GASF-CNN model, initially proposed by Sun and Ren.^29^ We systematically explored adjustments to various architectural components to optimize the model’s fault diagnosis capabilities. This detailed examination includes the rationale behind each modification and its implementation.

Our exploration focused on four specific types of modifications to assess and improve the performance change of the GASF-CNN approach:

1. Introducing an additional transformation to the matrices derived from the GASF.
2. Adjusting the kernel size of the initial convolutional layer.
3. Implementing hybrid approaches that combine the additional transformation and kernel size adjustments based on preliminary results.
4. Evaluating the impact of varying the number of filters across the convolutional layers.

#### Modification of Type 1

The original approach following the GASF transformation involves stacking the resulting matrices to serve as input channels for the CNN. The model processes the stacked matrices of information within the observation window in localized regions determined by the kernel size of the convolutional layers. Consequently, relationships involving time samples separated by longer differences than the kernel size are typically captured only in deeper layers of the network. To address this limitation, we explored a modification to enrich the data from the outset.

**Figure 6:**
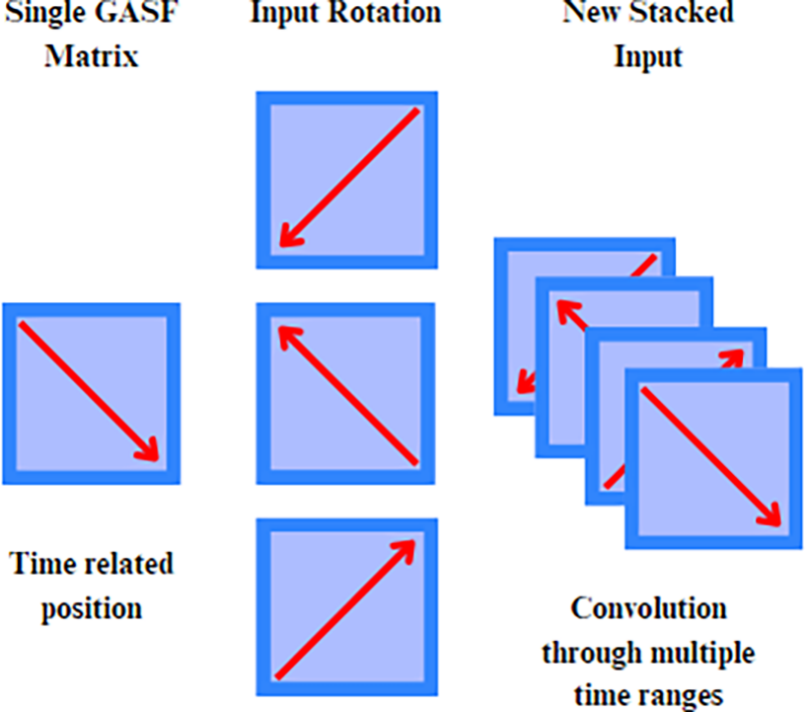
Illustration of the type 1 investigation strategy.

Specifically, we investigated the impact of enabling the simultaneous processing of time samples beyond the kernel size starting from the first layer.Our approach introduces additional transformations to the GASF matrices by generating rotated versions at angles of 90, 180, and 270°. These rotations present the model with alternative perspectives of the data’s temporal and spatial relationships, enabling it to consider broader patterns during its initial layers. As illustrated in Figure 6. This transformation, applied within the custom GASF layer, has the potential to enrich the input data from the beginning, allowing the model to process more comprehensive temporal information right from the initial processing stages.

#### Modifications of Type 2

Given the time-position structure of the GASF matrices, we investigated modifying the kernel size in the first convolutional layer. In the original model, using a 1 × 1 kernel limits the receptive field of the first layer, preventing it from capturing interactions between neighboring elements. This restriction could hinder the model’s ability to fully exploit the spatial and temporal relationships encoded in the GASF matrices, particularly at the early stages of feature extraction, especially because of the number of channels.

In order to address this limitation, we replaced the 1 × 1 kernel with larger kernels, specifically 3 × 3 and 5 × 5, which allow the model to process information from a broader spatial and temporal context in the initial layers. This adjustment was motivated by the theoretical advantage of larger kernels in capturing local dependencies, as they can aggregate information from multiple neighboring elements, potentially leading to richer feature representations.

We also explored the impact of reducing the number of filters from 128 to 64. This adjustment aimed to mitigate the risk of overfitting, particularly when using larger kernels, which increase the number of parameters in the model. Reducing the filter count can balance the model’s complexity, making it more computationally efficient while preserving its ability to learn meaningful patterns. By evaluating different kernel sizes and filter counts, we sought to balance the model’s complexity and performance, ensuring that the architecture remained both computationally efficient and robust against overfitting tendencies.

#### Modifications of Type 3

We also explored the potential benefits of combining types 1 and 2 modifications, concurrently implementing the enhanced data transformation and the adjusted kernel sizes. By stacking multiple rotated copies of the GASF matrices (type 1 modification) and using larger convolutional kernels (type 2 modification), we aimed to enrich the model’s ability to capture and process spatial and temporal patterns.

The rationale behind this combined approach is that the increased data complexity from multiple GASF matrix rotations could overwhelm the point-wise convolution of the original model, limiting its effectiveness in extracting relevant features. Larger convolutional kernels might better capture the enriched data, as they can integrate information from a broader context within each layer. Thus, we decided to investigate the combined effects of these modifications aiming for a model capable of processing more comprehensive data representations and of effectively extracting features, potentially leading to improved overall performance.

#### Modifications of Type 4

Based on the results obtained from the previous experiments, we undertook a systematic investigation into the impact of varying the number of kernels in the convolutional layers to assess its effect on model performance. This modification was motivated by the observation that the original architecture, with its fixed filter counts, might not fully capture the complexity of the temporal and spatial patterns within the GASF matrices. By systematically increasing the number of filters, we aimed to investigate the enhancement of the model’s capacity for feature extraction in layers processing lower and higher-level representations.

For this exploration, we incrementally increased the filter count across different layers, as detailed in Table 2.

**Table 2:** Topology for the Investigations of Type 4

Model ID	amount of filters in convolutional layer					
	conv 1	conv 2	conv 3	conv 4	conv 5	conv 6
Model 1	64	128	128	128	256	N/A
Model 2	128	128	128	128	256	N/A
Model 3	256	128	128	128	256	N/A
Model 4	512	128	128	128	256	N/A
Model 5	1024	128	128	128	256	N/A
Model 6	2048	128	128	128	256	N/A
Model 7	1024	512	128	128	256	N/A
Model 8	1024	512	512	128	256	N/A
Model 9	1024	512	512	256	256	N/A
Model 10	1024	512	512	256	256	128
Model 11	1024	512	512	256	256	256

It is important to note that we consistently set the kernel size of the initial convolutional layer to 3 × 3 during these investigations due to improvements observed from modifications of type 2. Additionally, two of the evaluated configurations included an extra convolutional layer. This addition was designed to investigate whether increased depth would enhance the model’s ability to extract features and process complex patterns effectively.

The primary theoretical motivation for these modifications is that increasing the number of filters in the convolutional layers provides a greater capacity to learn diverse features from the input data. Additionally, adding depth to the network could facilitate hierarchical feature extraction, where subsequent layers build upon the representations learned by earlier ones.

For clarity and ease of reference, we only assigned specific model IDs to models resulting from modifications of type 4. This labeling approach facilitates a straightforward comparison of results and supports a clear discussion of the findings in subsequent sections.

### Evaluation Metrics

Given that fault diagnosis in the Tennessee Eastman Process (TEP) is a multiclass classification task, we use the F1-score and confusion matrices as our primary evaluation metrics. The F1-score is calculated for each class to measure the model’s precision and recall balance. It is computed using the expressions shown in eq 4.
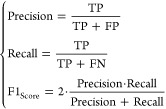
4

In these equations, TP denotes true positives, FP represents false positives, and FN refers to false negatives.

To summarize overall model performance across all classes, we use the macro-averaged F1-score. This metric consists of the average of the F1-scores for each class, providing a balanced view of the model’s performance on both majority and minority classes.

We also compare our results with those reported in the Literature to contextualize our model’s performance. Previous studies, such as those by Sun and Ren,^29^ have reported F1-scores and confusion matrices for similar multiclass classification tasks, allowing us to benchmark our findings against established methods.

## Results and Discussion

In this section, we present and discuss the results of our investigation. We start by examining the impact of the learning rate on the baseline GASF-CNN model and identifying the optimal configuration. Next, we provide detailed results for the best baseline model, followed by evaluating the four types of modifications that enhance fault diagnosis. It is important to note that we prioritized keeping only the key training curves in the article’s main body. We reported the entire collection of training curves of the investigated modifications as Supporting Information. Finally, we compare our findings with those reported in the Literature to contextualize our results.

### Impact of Learning Rate on Baseline Model Performance

**Figure 7:**
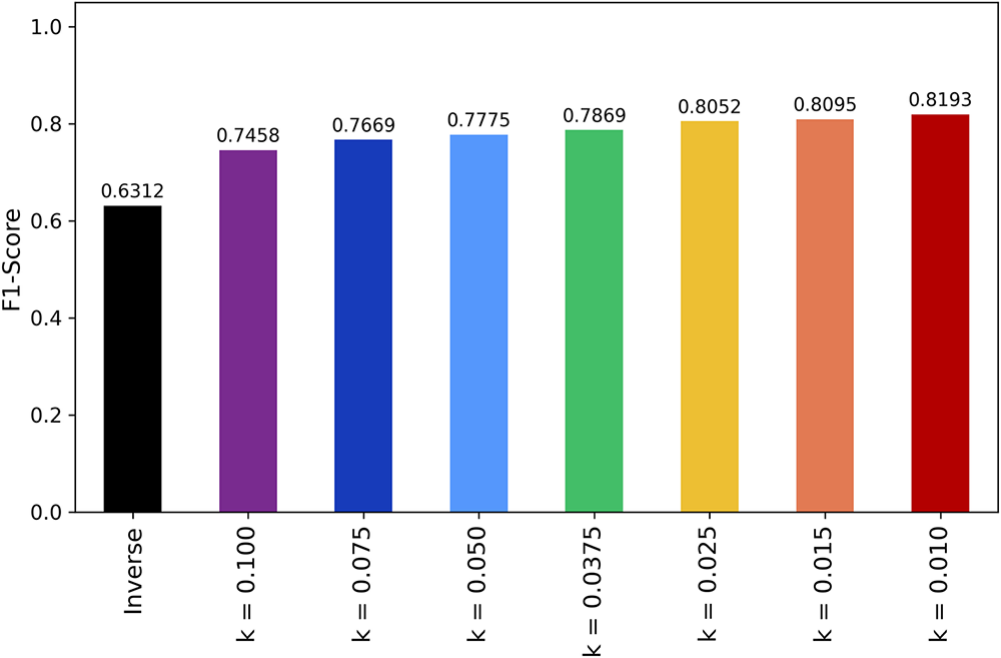
Learning rate decay functions results.

The results reveal that the baseline model is sensitive to the choice of learning rate decay. Figure 7 illustrates the varying average validation F1-scores obtained under different learning rate decay strategies.

Since the exponential decay function set at a rate of *k* = 0.01 demonstrated the best validation performance (81.93%), we adopted this configuration as the baseline for evaluating subsequent modifications. This choice ensures that all further investigations are conducted under optimal learning conditions, providing a consistent reference point for comparison.

**Figure 8:**
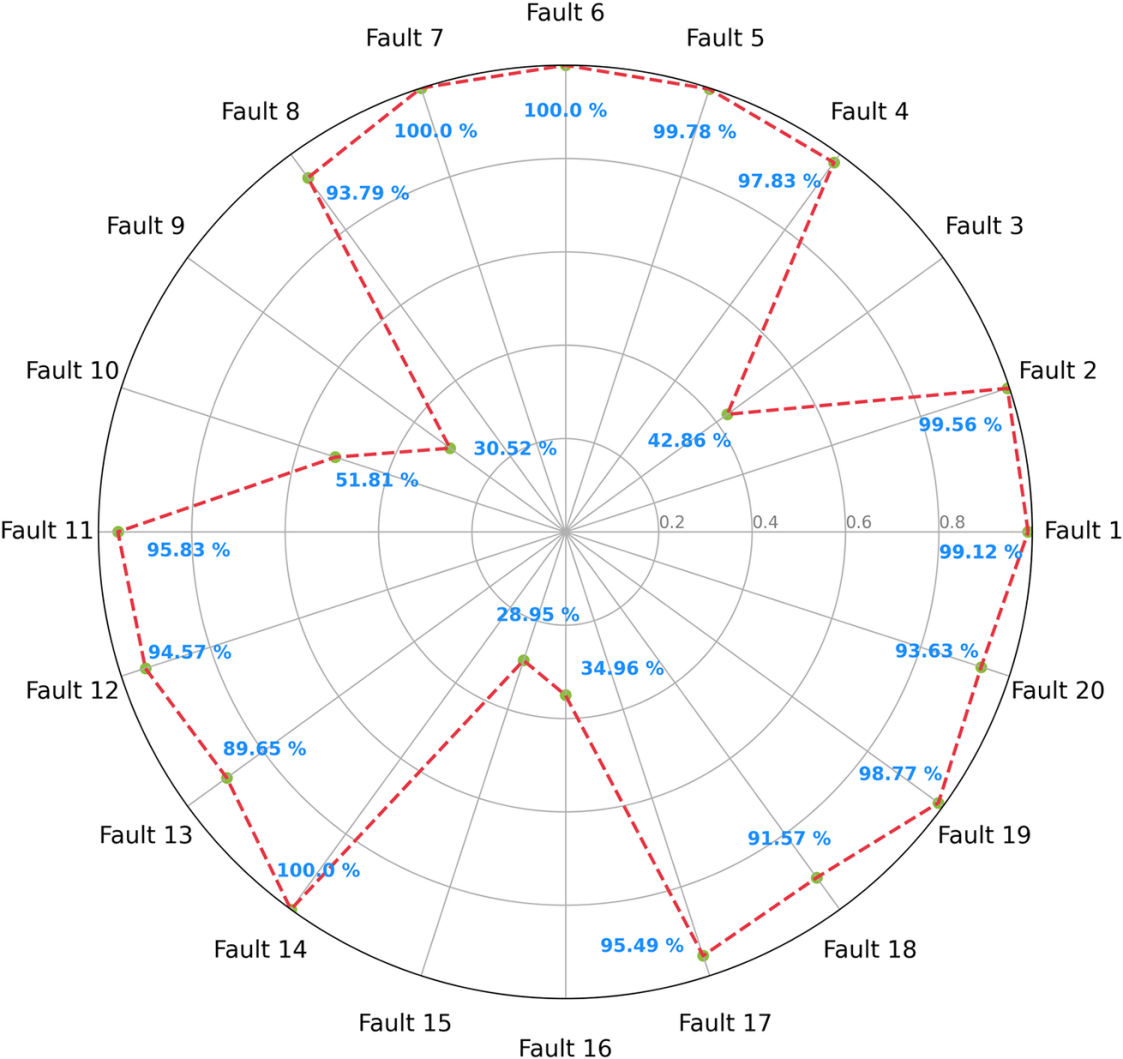
Validation F1-scores for the baseline model.

**Figure 9:**
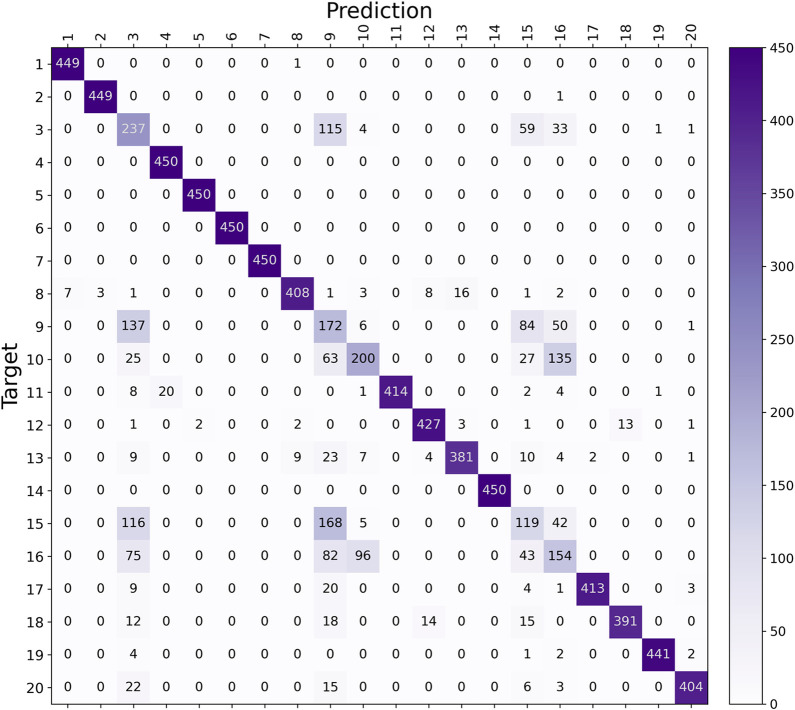
Validation confusion matrix for the Baseline Model.

We conducted a comprehensive analysis to gain deeper insights into the baseline model’s performance. Figure 8 details the validation F1-scores for each fault class, offering a nuanced view of the model’s ability to differentiate between various fault conditions. Complementing this, Figure 9 presents the confusion matrix, further explaining how the model misclassifies different faults.

The model demonstrates its high capability of classifying 15 of the 20 faults, staying above 89% F1-scores. However, the model faces challenges with faults 3, 9, 10, 15, and 16, where the F1-scores are below 52%. These results suggest difficulties in distinguishing these faults from others, possibly due to their physical nature. Apart from fault 16, which is of an unknown type, all other faults in this group are related to feed temperature or heat transfer.

The results from the confusion matrix shown in Figure 9 further reinforce the hypothesis for the model’s misclassification cases. It indicates that these specific faults are frequently confused with each other, indicating that their similar physical nature plays an important role in distinguishing them.

**Figure 10:**
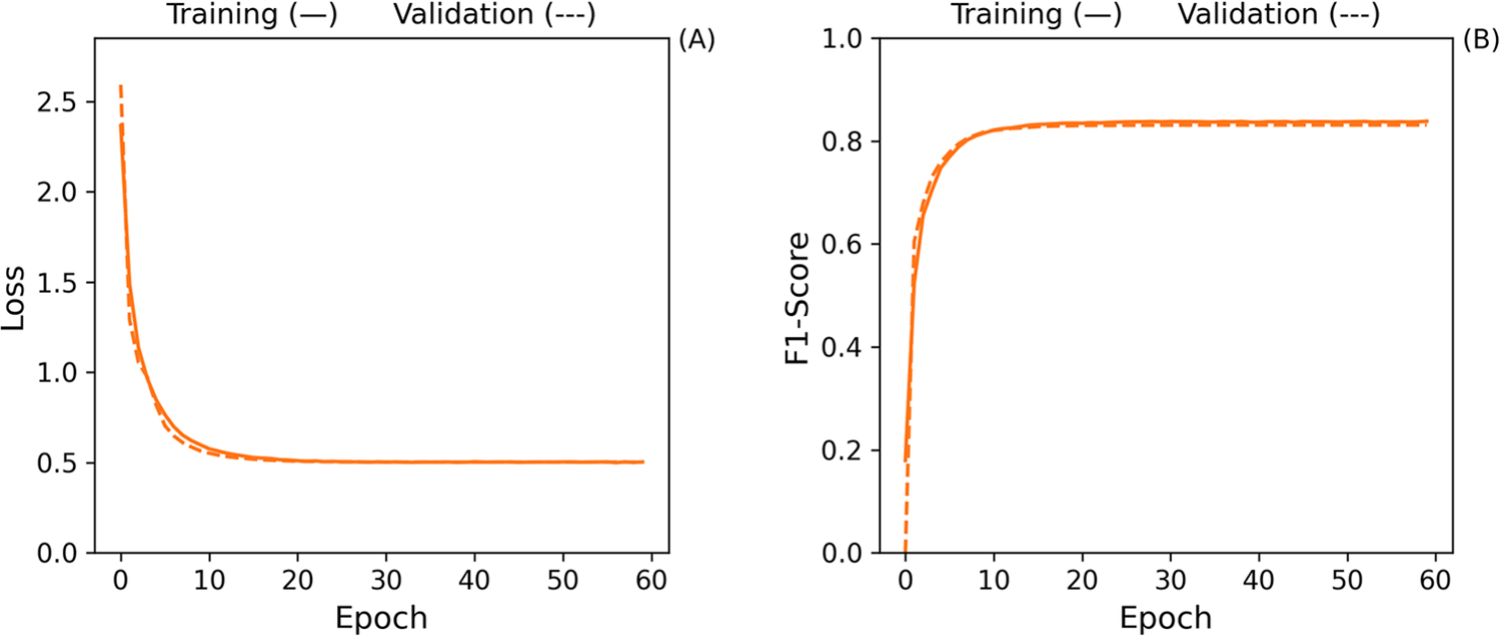
Loss (A) and F1-score (B) training curves for the Baseline Model.

An examination of the loss curve shown in Figure 10 reveals no signs of overfitting in the model. The loss decreases consistently throughout training, indicating stable learning without excessive divergence between training and validation data.

### Influence of the Investigated Modifications

We designed the modification of type 1 to investigate the hypothesis that simultaneously processing time lags further than kernel size, which could benefit model learning. It consisted of stacking the original GASF matrices with copies rotated by 90, 180, and 270°.

This modification resulted in slightly lower performance than the baseline model, with an average F1-score of 81.13%. This result suggests that while the intention was to provide the model with more comprehensive temporal information, the rotated layers did not enhance, and may have even reduced, the model’s ability to generalize. Adding rotated matrices may have introduced complexity or noise that outweighed the benefits of increased temporal coverage.

The modifications of type 2 focused on addressing a potential bottleneck in the baseline model’s architecture, specifically in the first convolutional layer due to the filter quantity and point-wise convolutions. It involved experimenting with filter quantities of 64 and 128, using both 3 × 3 and 5 × 5 kernels. The results presented in Table 3 show that increasing the size of the kernels in the first convolutional layer led to improved classification performance.

**Table 3:** Average Validation F1-Scores for Modifications of Type 2

parameters	kernel 3 × 3	kernel 5 × 5
64 Filters	82.35%	82.59%
128 Filters	82.93%	82.68%

The results indicate that reducing the number of filters in the initial convolutional layer negatively impacted classification performance. Larger filter sizes, particularly the 3 × 3 kernels, improved the model’s ability to capture complex features. However, it is important to note that while increasing filters enhanced performance, other configurations constrained the benefit, evidenced by observing that the effects of changing the number of filters with 5 × 5 kernels were less impactful than in the 3 × 3 kernel cases, considering the current configuration.

Type 3 modifications aimed to explore potential synergistic effects by combining data augmentation with rotated layers. Specific results are shown in Table 4.

**Table 4:** Average Validation F1-Scores for Modifications of Type 3

parameters	kernel 3 × 3	kernel 5 × 5
64 Filters	81.51%	56.67%
128 Filters	82.54%	47.89%

Even with the increased filters, the combined approach of data enrichment through rotated layers did not yield significant improvements over the original GASF maps. For kernel size 5 × 5, a noticeable performance drop occurred, possibly related to overfitting. These results suggest that while individual enhancements showed promise for type 2, the combined use with type 1 modification did not provide additional benefits and may have contributed to model instability. This outcome indicates that the enriched data from rotating the matrices did not improve the model’s fault classification capabilities and highlights the importance of evaluating the trade-offs between data complexity and model performance.

**Figure 11:**
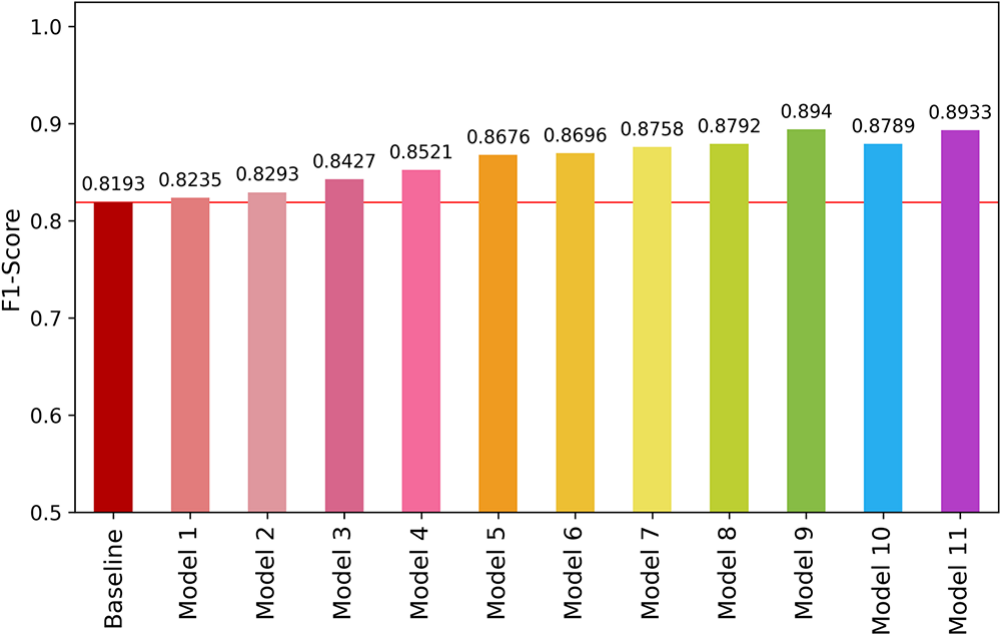
Validation average F1-score for type 4 modifications.

In light of the results, we considered the hypothesis that further increasing the number of filters in convolutional layers could enhance classification performance. It involved maintaining 3 × 3 kernels in the first convolutional layer and progressively increasing the number of filters throughout the convolutional layers. The average F1-score for each modification is shown in Figure 11, including the two 3 × 3 kernel size cases of type 2 modifications and cases of additional convolutional layers.

**Figure 12:**
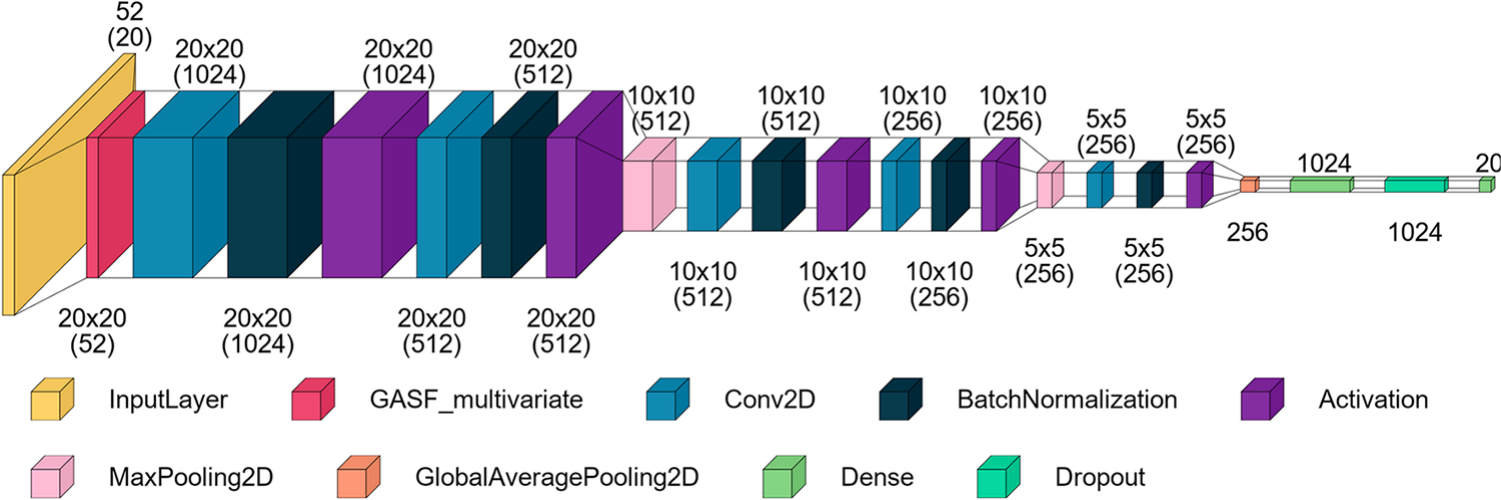
Representation of the architecture of Model 9.

The results indicate a significant improvement in classification performance with the progressive increase in the quantity of filters. Model 9 achieved a maximum F1-score of 89.40%, which is 7.47% higher than the baseline model. Its architecture is illustrated in Figure 12. This increase demonstrates that systematically augmenting the number of filters was a key factor in substantially enhancing model performance in our study.

**Figure 13:**
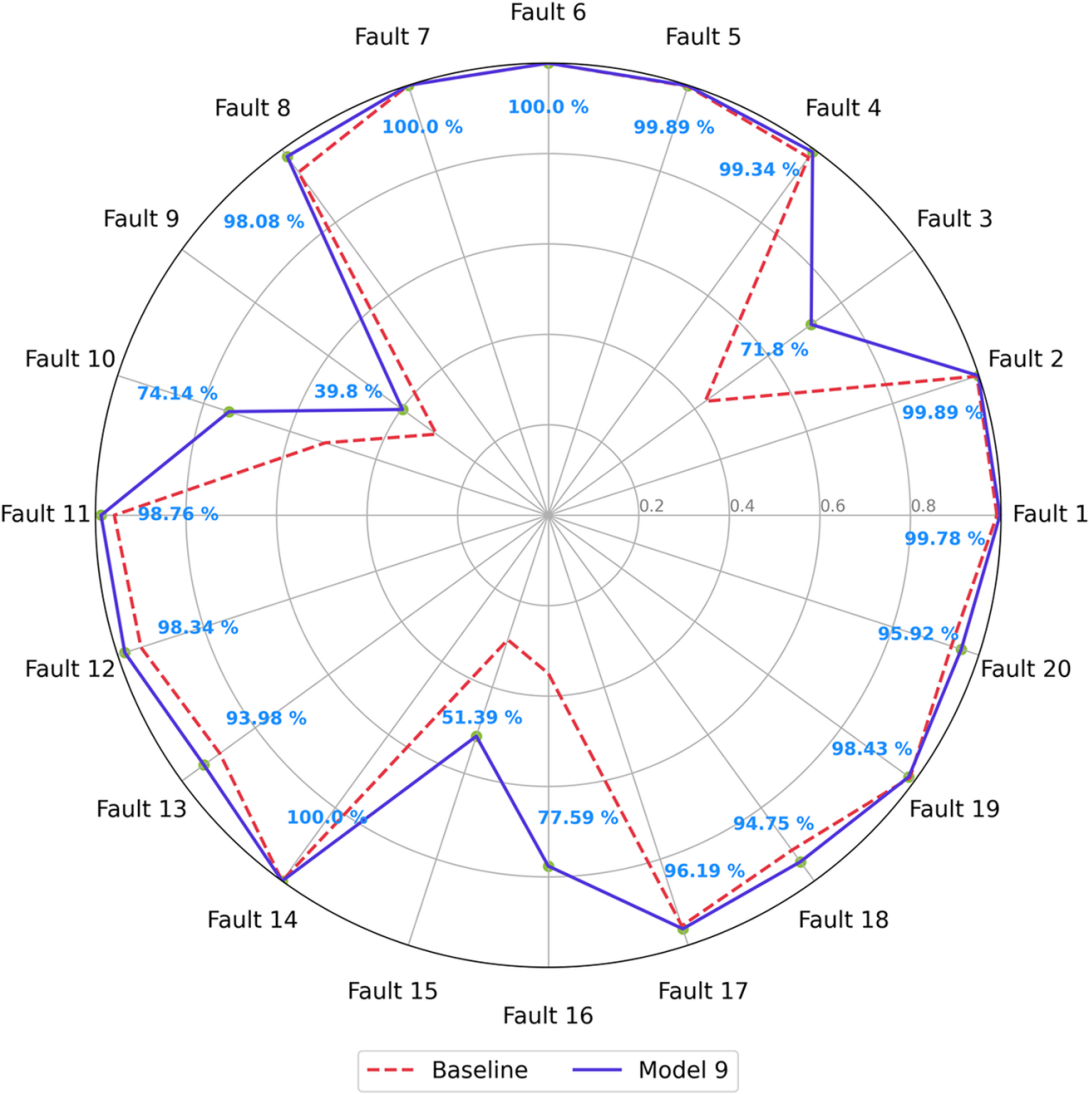
Validation F1-scores for Model 9.

Figure 13 displays a radar plot comparing the F1-scores for each fault class between Model 9 and the baseline model. It is possible to observe that Model 9 exhibited improved classification capabilities across all faults. The overall improvement in performance may be linked to the nature of the GASF input matrices, which are significantly more complex than typical image data. Fifty-two channels far surpass the common three channels used in standard image data. In this case, more kernels seem necessary to effectively capture and detect the more intricate and harder-to-find patterns within the data. The increase in kernel quantity allows the model to better process and extract relevant features from the extensive information embedded in the GASF matrices.

**Figure 14:**
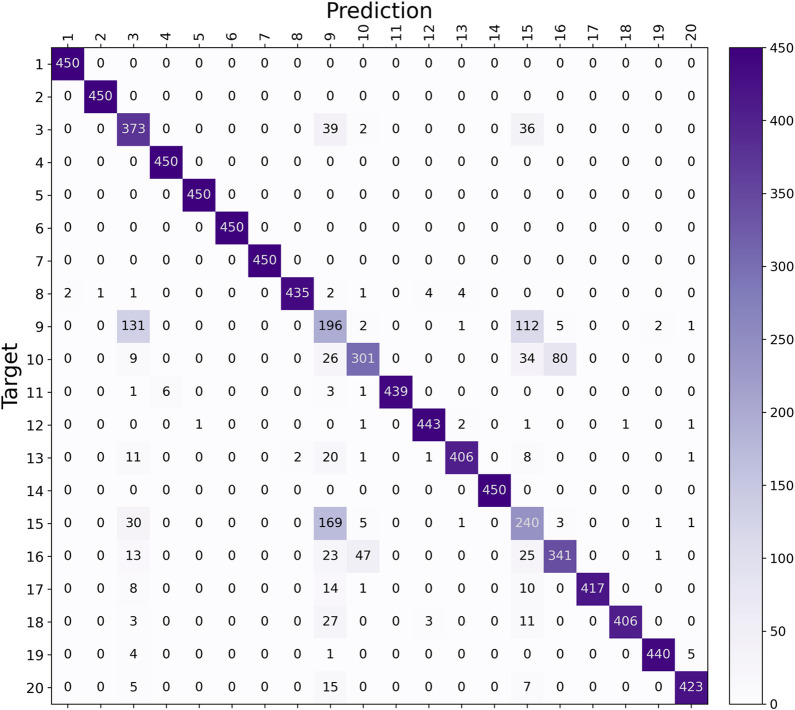
Validation confusion matrix for Model 9.

The model demonstrated significant improvement in predicting all five challenging faults, with enhancements of 28.94% for fault 3, 9.28% for fault 5, 21.29% for fault 10, 22.44% for fault 15, and 43.62% for fault 16. As shown in the confusion matrix in Figure 14, misclassification among these faults is less widespread. However, faults 3, 9, and 15 remain challenging to distinguish, especially for faults 3 and 9, which are both temperature-related issues on the same feed stream, making them inherently more complex to separate. Additionally, while faults 10 and 16 are frequently confused with each other, they are also closely linked to the misclassification of faults 3, 9, and 15.

**Figure 15:**
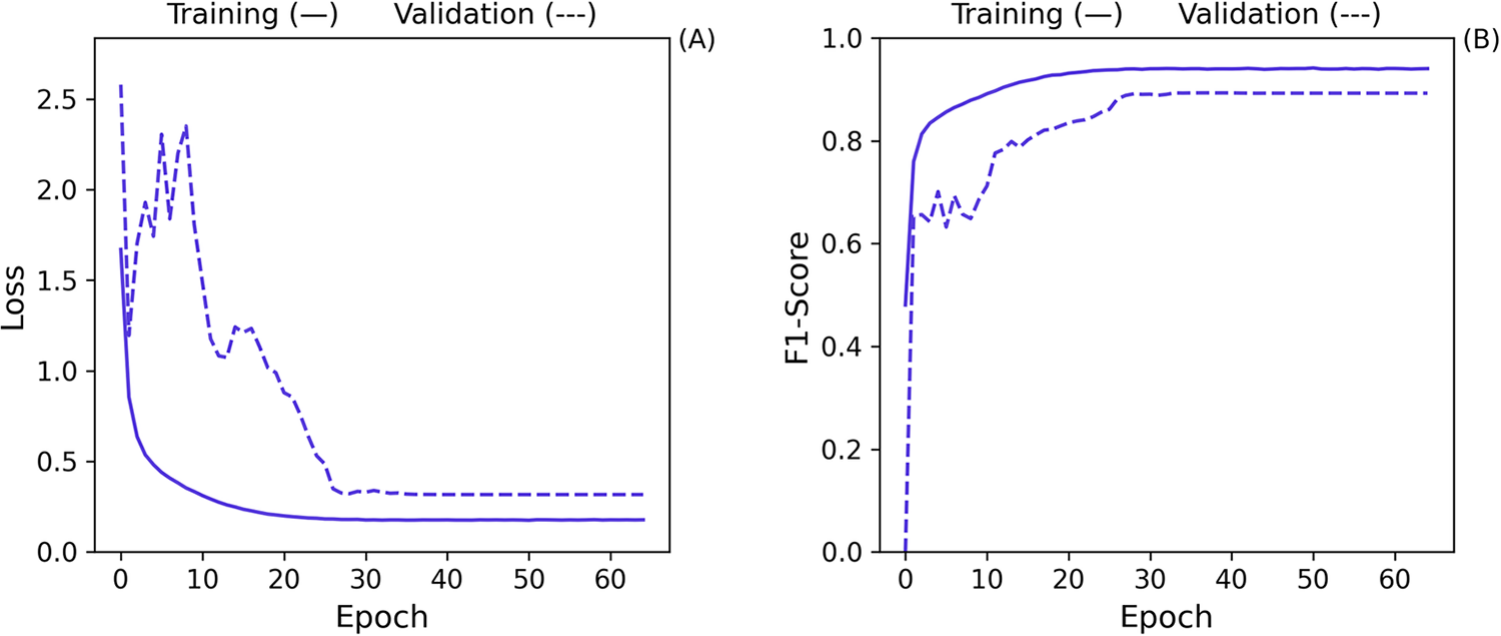
Loss (A) and F1-score (B) training curves for the best model.

Figure 15 illustrates the training curves, where the validation loss initially exhibits some volatility but eventually stabilizes as training progresses. While the validation loss curve did not exactly converge with the training loss, this is not a concern because the model’s performance on unseen data remained robust. Early stopping was activated, indicating the model achieved optimal performance without overfitting.

### Testing Results and Comparative Analysis with the Literature

**Figure 16:**
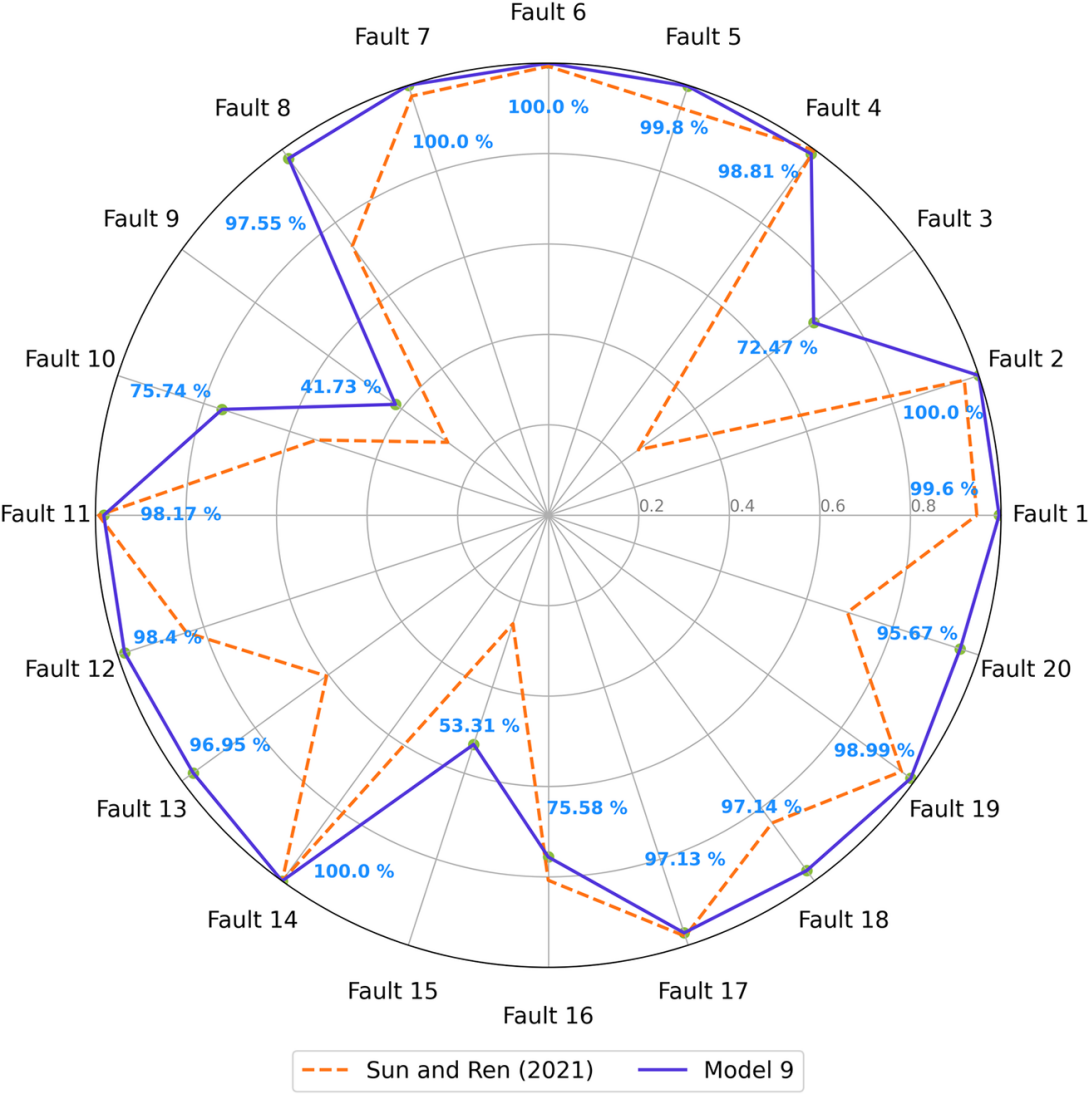
Test F1-scores for Model 9 and the reference GASF-CNN.^29^

The performance of Model 9 on the testing data set is illustrated in Figure 16. The F1-scores for each fault class demonstrate that the model’s effectiveness extends beyond the validation set.

The average F1-score for the test data set is 89.85%, showing only a negligible difference from the validation average. This minimal decrease suggests that Model 9 generalizes effectively to new, unseen data, maintaining consistent performance. Compared to the results reported by Sun and Ren^29^ for their GASF-CNN model, which served as the basis for our architecture, Model 9 demonstrates comparable or even significantly improved performance in fault diagnosis of the Tennessee Eastman Process, except for fault 16. Additionally, Model 9 achieved an average F1-score that is 11.86% higher than the 77.99% reported by Sun and Ren.^29^

For a broader perspective, in Table 5, we compare Model 9’s performance against other state-of-the-art models from the literature. While Model 9 demonstrates competitive F1-scores across most fault classes, it notably outperforms Fault 18 with F1-scores of 97.14%. However, the performance in Fault 9 and Fault 15 suggests room for further refinement, as indicated by the lower scores compared to Tao et al.^46^

**Table 5:** Model 9 Test F1-Scores Comparison with State-ofc-Art Works from the Literature

class	Model 9	ref (1)^a^	ref (2)^b^	ref (3)^c^	ref (4)^d^
Fault 1	99.60%	94.67%	100.0%	99.81%	100.0%
Fault 2	100.0%	96.63%	100.0%	99.40%	99.80%
Fault 3	72.47%	24.62%	not informed	96.98%	58.00%
Fault 4	98.81%	99.87%	99.00%	99.97%	100.0%
Fault 5	99.80%	94.67%	89.00%	93.87%	80.30%
Fault 6	100.0%	99.30%	90.00%	99.46%	100.0%
Fault 7	100.0%	97.50%	99.00%	100.0%	100.0%
Fault 8	97.55%	73.61%	82.00%	98.45%	75.40%
Fault 9	41.73%	27.45%	not informed	87.59%	0.00%
Fault 10	75.74%	53.84%	77.00%	98.61%	94.20%
Fault 11	98.17%	99.29%	99.00%	99.40%	99.90%
Fault 12	98.40%	83.99%	83.00%	97.83%	86.70%
Fault 13	96.95%	60.52%	57.00%	98.41%	87.80%
Fault 14	100.0%	99.94%	100.0%	99.62%	85.80%
Fault 15	53.31%	25.22%	not informed	83.50%	0.40%
Fault 16	75.58%	80.74%	77.00%	not informed	1.00%
Fault 17	97.13%	97.92%	99.00%	not informed	97.90%
Fault 18	97.14%	84.13%	81.00%	not informed	84.20%
Fault 19	98.99%	96.42%	99.00%	not informed	99.80%
Fault 20	95.67%	69.49%	95.00%	not informed	98.00%
Average F1-score	89.85%	77.99%	N/A	N/A	77.46%

a Sun and Ren.^29^

b Ren et al.^47^

c Tao et al.^46^

d E Souza et al.^45^

It is also worth noting that some references do not include results for all fault classes, as observed in Ren et al.’s^47^ and Tao et al.’s^46^ work. It allows models to become more specialized at classifying the subgroup of faults at the expense of not covering the original 20 faults. Additionally, the mentioned studies that specified observation window sizes used windows of similar or larger duration. Sun and Ren^47^ initially investigated window lengths between 10 and 40. They developed the remaining of their study with lengths of 35 and 40. Tao et al.^46^ have set a time length of 20 as the input of their CNN-1D, which we calculated from the output dimensions and the architecture configurations. E Souza et al.^45^ chose a window size of 52 to maintain a square input matrix, which further emphasizes the improvement of our approach since less historical data is necessary to provide a diagnosis.

Overall, the consistent and high F1-scores across various faults suggest that Model 9 offers a reliable and effective approach to fault diagnosis, with potential advantages over existing methods documented in the literature.

Although we obtained a generalized enhanced performance with the current state of our research, there is still space for improvement. Further modifications within the realm of CNNs can still be explored for further performance enhancement. Besides, our investigation has primarily focused on model diagnosis, leaving the detection aspect of FDD as an area open for exploration in future investigations.

## Conclusions

In this study, we developed and evaluated a convolutional neural network architecture for fault detection and diagnosis in the Tennessee Eastman Process. Our approach used Gramian Angular Summation Fields to transform multivariate time series data into 2D images, allowing the CNN to extract complex spatiotemporal patterns. The results demonstrated that Model 9 achieved an average F1-score of 89.85% on the testing data set, which remained consistent with the validation results, indicating strong generalization capabilities.

Compared to the GASF-CNN model by Sun and Ren,^29^ which served as the foundation for our architecture, Model 9 showed comparable or improved performance across most faults, particularly in diagnosing some of the more challenging faults in the TEP. The change in the number of kernels was a vital parameter to achieve performance improvement, which allowed the model to capture more nuanced patterns in the data, especially given the complexity introduced by the 52-channel GASF matrices.

Our focused approach to refining a single architecture, rather than exploring multiple variations, has proven effective in identifying and optimizing the key aspects of the model that directly impact its performance. This methodical focus allowed us to gain deeper insights into the architecture’s specific strengths and weaknesses, leading to targeted improvements.

While our research has achieved an improved performance, there is still room for further enhancement. Future works could explore additional modifications within the CNN framework, such as experimenting with different hyperparameters and other input transformations or even integrating advanced techniques like attention mechanisms to boost performance further. Although we focused our investigation on the TEP data set due to its industrial relevance and complexity, it would be relevant to explore how our approach holds up against other data sets in future works. Moreover, since our investigation primarily focused on fault diagnosis, future studies could expand on the detection aspect of the problem, exploring the full spectrum of FDD to develop a more comprehensive solution.

## Supporting Information Available

Training curves of investigated modifications (PDF)
