# Supplementary material for: Can Focusing on One Deep Learning Architecture Improve Fault Diagnosis Performance?
Source: J Chem Inf Model. 2025 Jan 30;65(3):1289–304. doi: 10.1021/acs.jcim.4c02060 (PMC11815845; doi:10.1021/acs.jcim.4c02060)
Supplement: Supplementary file 1 — ci4c02060_si_001.pdf [file ci4c02060_si_001.pdf]

Supporting Information

Can Focusing on One Deep Learning Architecture Improve Fault Diagnosis Performance?

*João G. Neto[1], Karla Figueiredo[2], João B. P. Soares[3], Amanda L. T. Brandão[1],**

[1]Department of Chemical and Materials Engineering, Pontifical Catholic University of Rio de Janeiro, 225, Marquês de São Vicente Street, Gávea, Rio de Janeiro, RJ, Brazil, 22451-900.

[2]Department of Computer Science, Rio de Janeiro State University, 524, Rector João Lyra Filho Pavilion, 6th floor, Maracanã, Rio de Janeiro, RJ, Brazil, 20550-013.

[3]Department of Chemical Engineering, University of Alberta, 9211, 116 Street, Edmonton, Alberta, Canada, T6G 1H9.

*Corresponding Author

Email: amanda.lemette@puc-rio.br

Supplementary Figures - Training Curves of Investigated Modifications

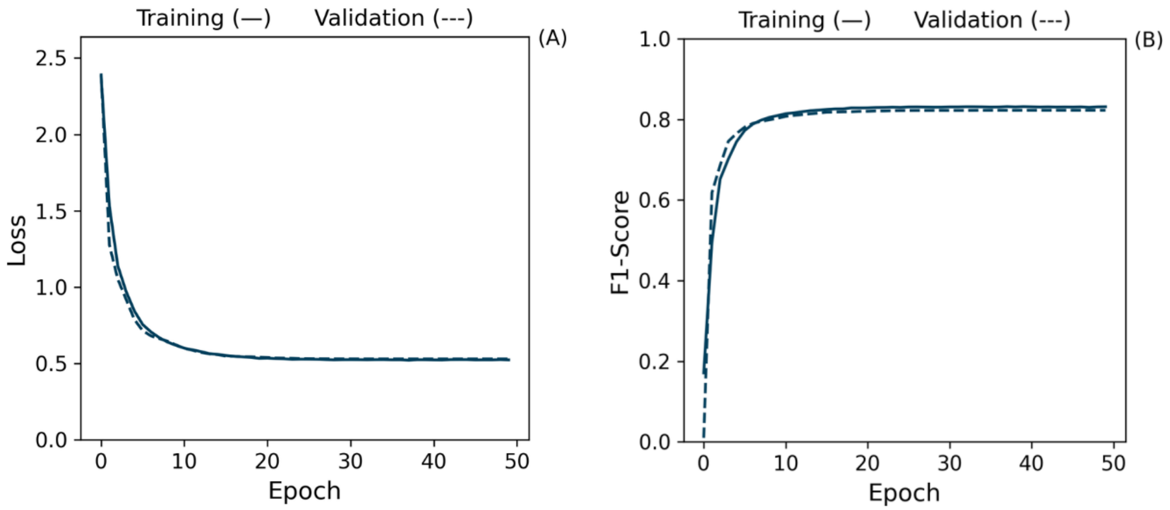

Figure S1. Loss (A) and F1-Score (B) training curves for modification of type 1.

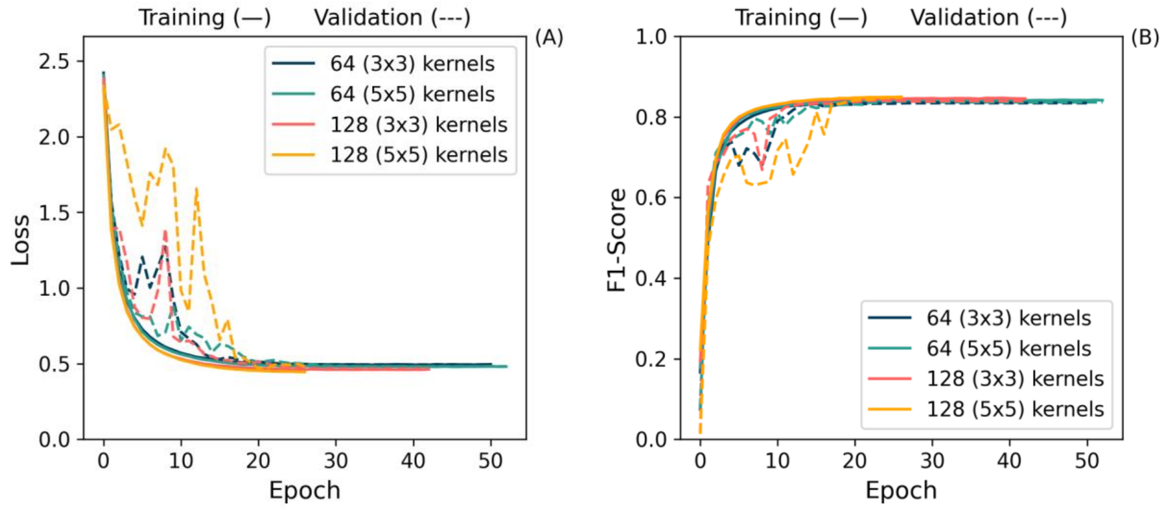

Figure S2. Loss (A) and F1-Score (B) training curves for modifications of type 2.

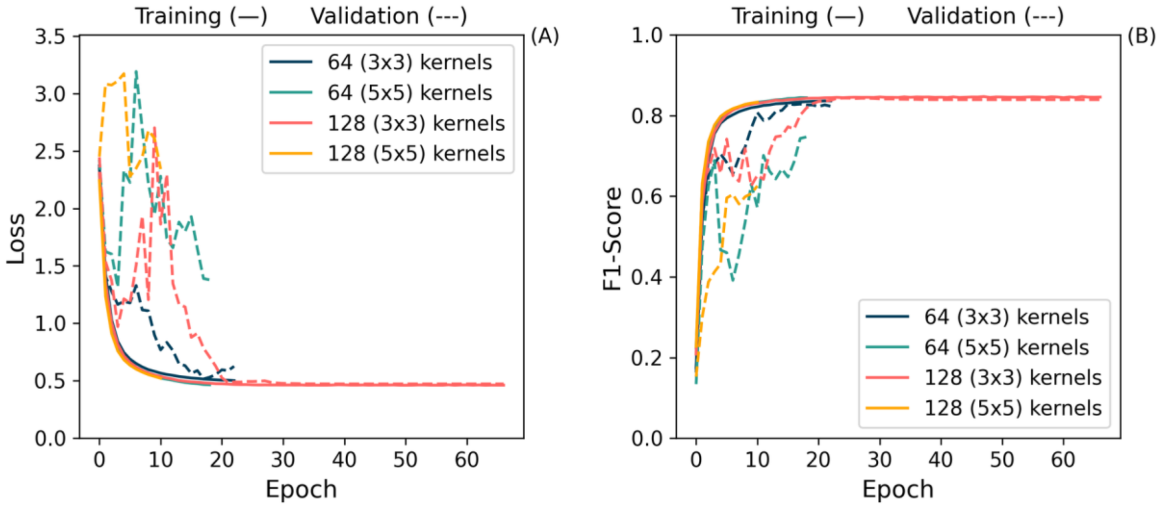

Figure S3. Loss (A) and F1-Score (B) training curves for modifications of type 3.

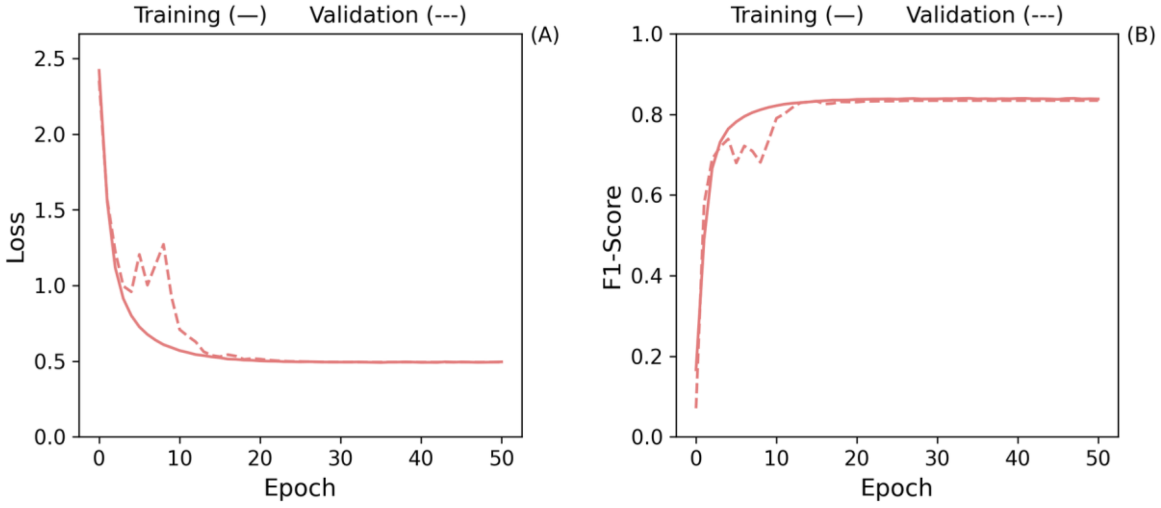

Figure S4. Loss (A) and F1-Score (B) training curves for Model 1.

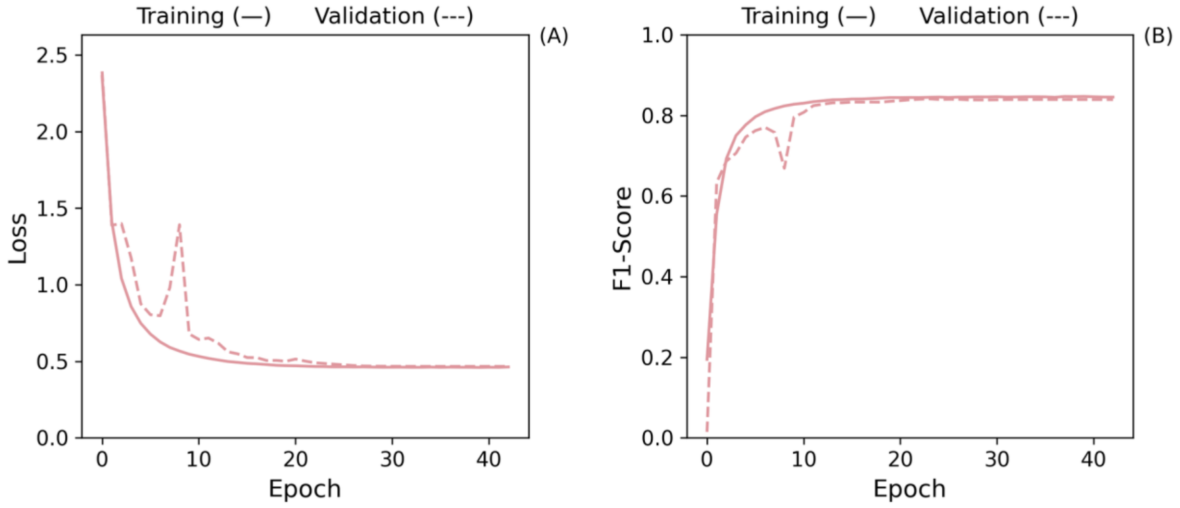

Figure S5. Loss (A) and F1-Score (B) training curves for Model 2.

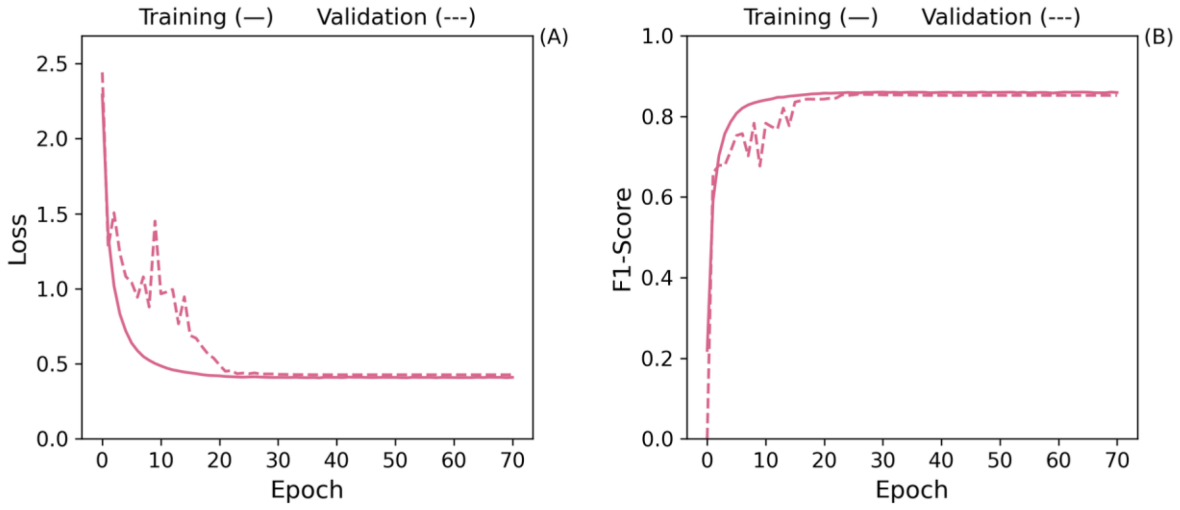

Figure S6. Loss (A) and F1-Score (B) training curves for Model 3.

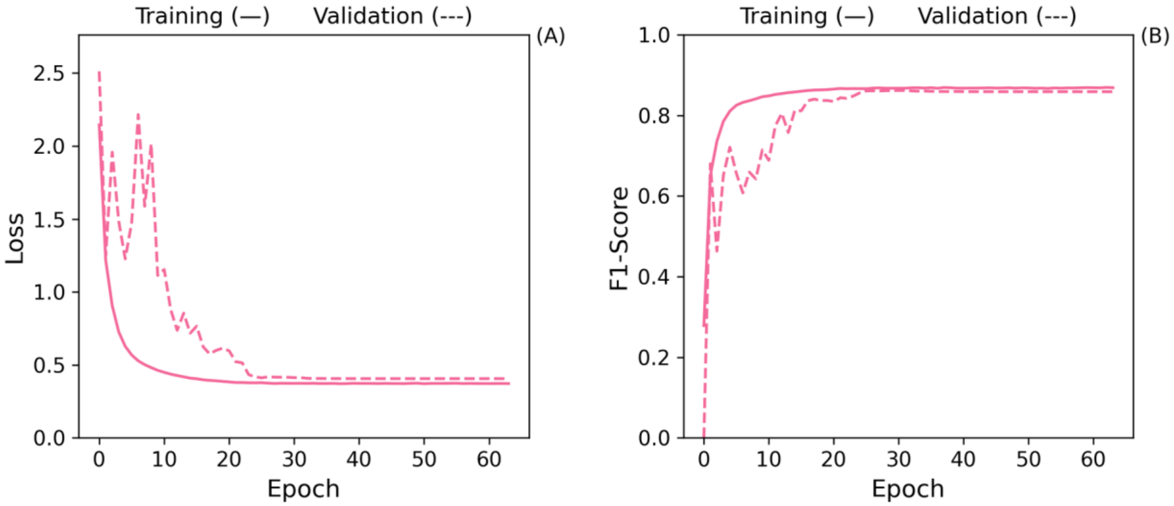

Figure S7. Loss (A) and F1-Score (B) training curves for Model 4.

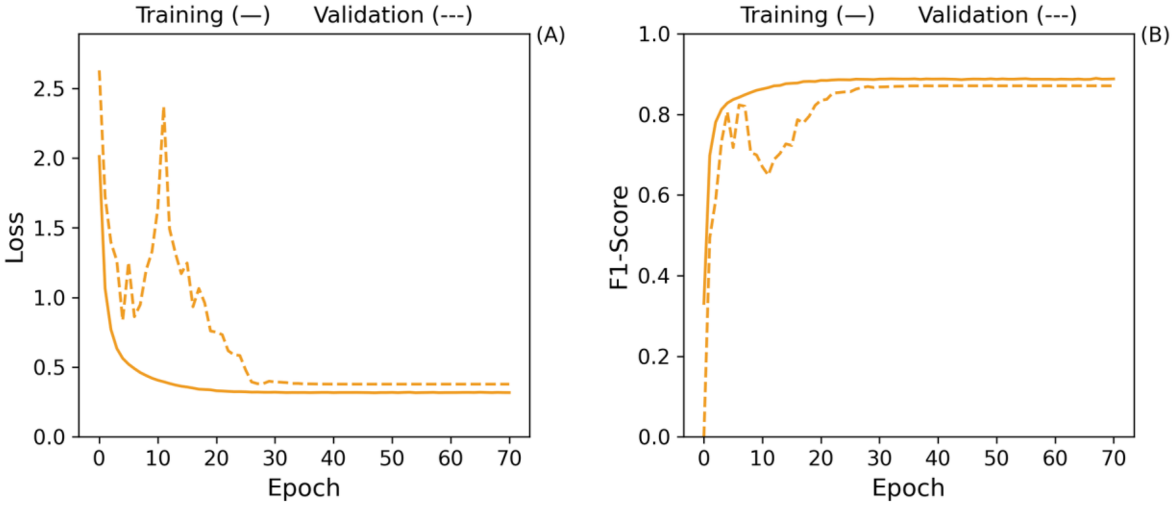

Figure S8. Loss (A) and F1-Score (B) training curves for Model 5.

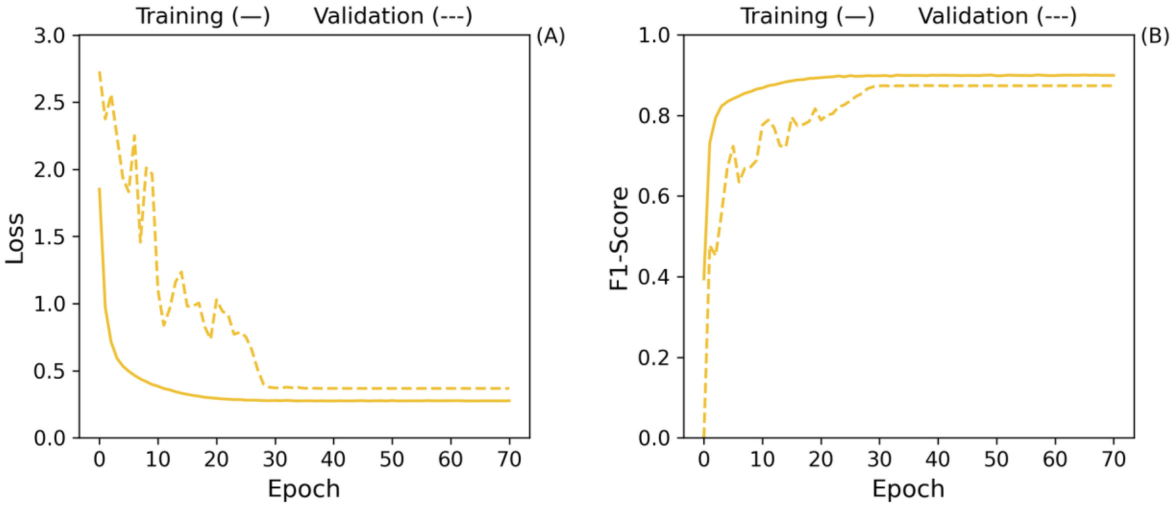

Figure S9. Loss (A) and F1-Score (B) training curves for Model 6.

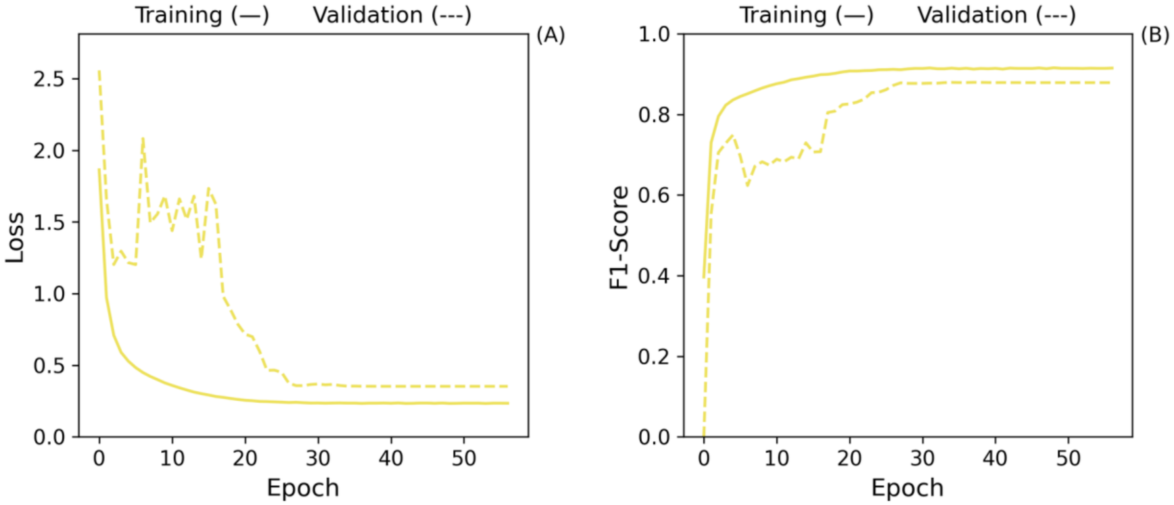

Figure S10. Loss (A) and F1-Score (B) training curves for Model 7.

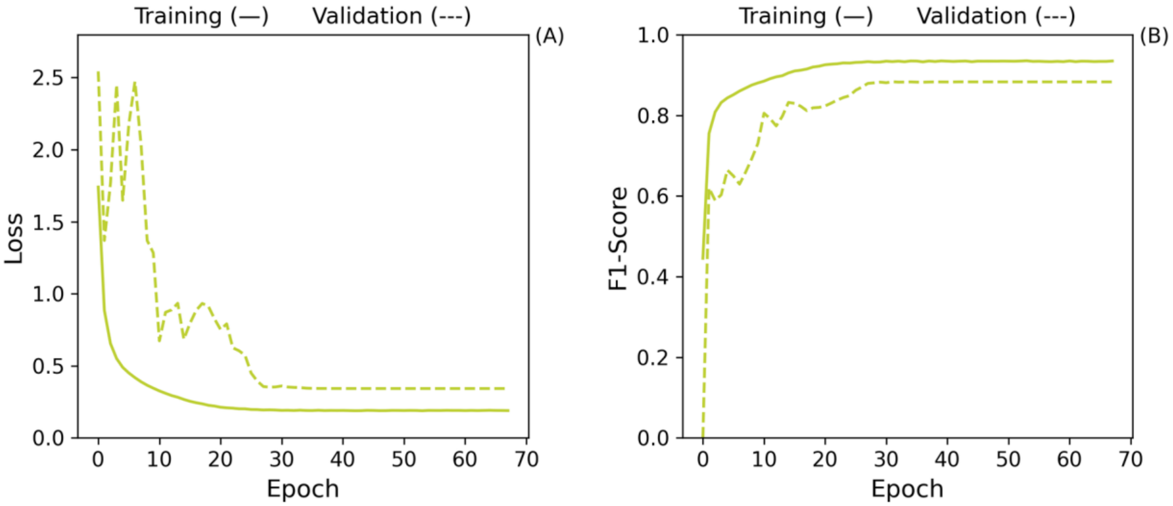

Figure S11. Loss (A) and F1-Score (B) training curves for Model 8.

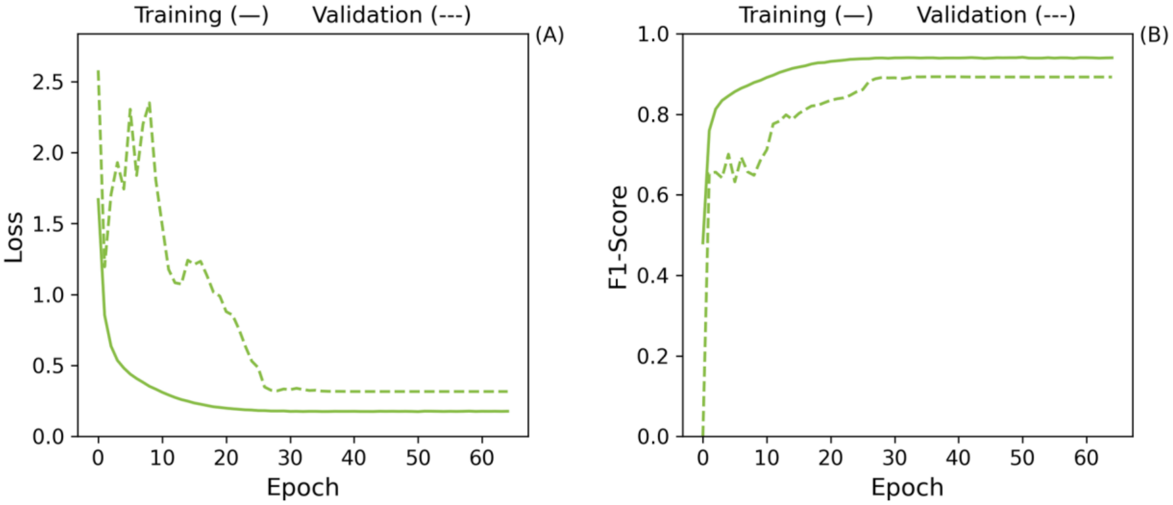

Figure S12. Loss (A) and F1-Score (B) training curves for Model 9.

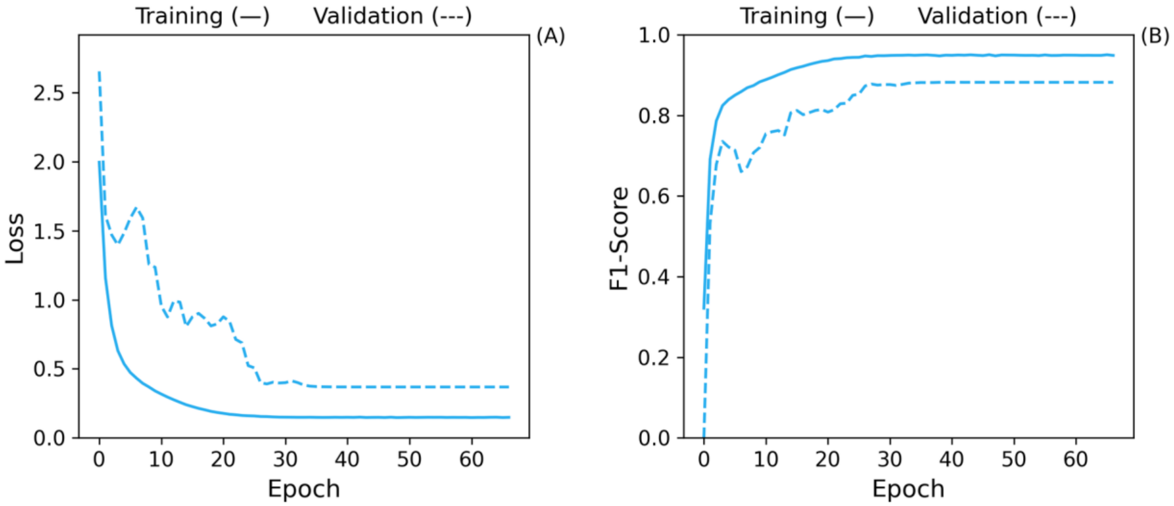

Figure S13. Loss (A) and F1-Score (B) training curves for Model 10.

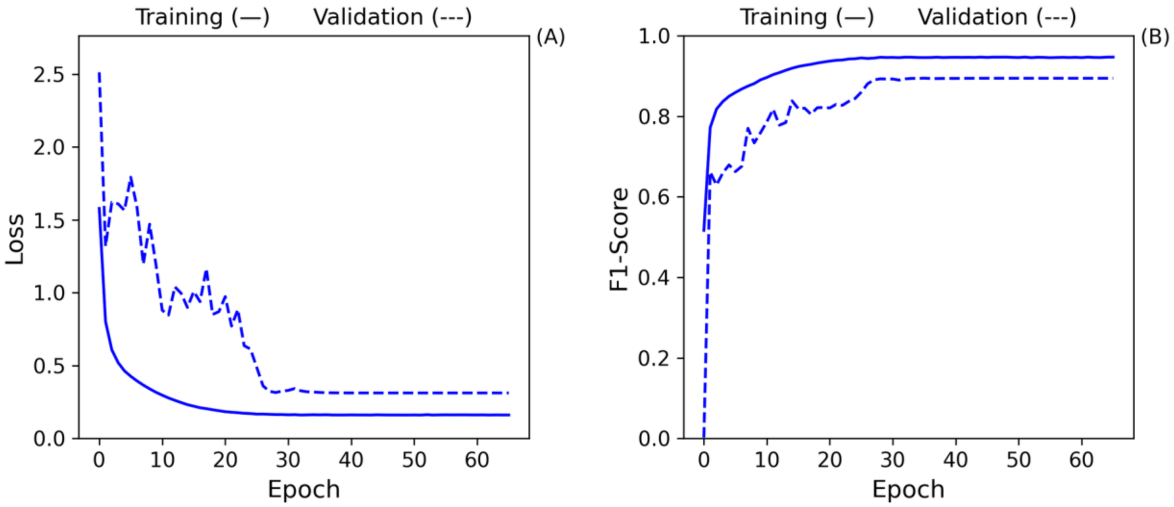

Figure S14. Loss (A) and F1-Score (B) training curves for Model 11.
